# Stratification of brain-derived extracellular vesicles of Alzheimer’s disease patients indicates a unique proteomic content and a higher seeding capacity of small extracellular vesicles

**DOI:** 10.1186/s40035-025-00519-z

**Published:** 2025-12-05

**Authors:** Marie Oosterlynck, Elodie Leroux, Balasubramaniam Namasivayam, Thomas Bouillet, Raphaelle Caillierez, Anne Loyens, Daniele Mazur, Romain Perbet, Christophe Lefebvre, Soulaimane Aboulouard, Claude-Alain Maurage, Bertrand Accart, Luc Buée, Morvane Colin

**Affiliations:** 1 Inserm UMR-S 1172, ‘Alzheimer & Tauopathies’, CHU-Lille, Lille Neuroscience & Cognition, Faculty of Medecine – pole recherche, University of Lille, Bâtiment Biserte, Rue Polonovski, 59000 Lille, France; 2 Inserm, U1192-Laboratoire Protéomique, Réponse Inflammatoire Et Spectrométrie de Masse (PRISM), University of Lille, Lille, France; 3 CHU-Lille, CRB/CIC1403, Centre de Ressources Biologiques du Centre d’Investigation Clinique, University of Lille, Lille, France

**Keywords:** Alzheimer's disease, Extracellular vesicles, Collagenase brain dissociation, Proteomic profiling, GWAS, FERMT2, Clusterin, Tau seeding

## Abstract

**Background:**

Alzheimer’s disease (AD) is the most prominent form of dementia worldwide. It is characterized by tau lesions that spread throughout the brain in a spatio-temporal manner. This has led to the prion-like propagation hypothesis implicating a transfer of pathological tau seeds from cell to cell. Human brain-derived extracellular vesicles (BD-EVs) isolated from the brain-derived fluid of AD patients contain seeds that contribute to this tau pathology spreading. Knowing the rich diversity of EVs, isolation of functional EV sub-populations is required to unravel their implication in the pathophysiology of AD.

**Methods:**

Here, enriched-small EVs (eSEVs) and enriched-large EVs (eLEVs) were isolated from frozen tissues after collagenase enzymatic brain dissociation to guarantee the best EVs’ integrity. Then proteomic profiling and tau seeding capacity testing were performed in vitro and in vivo.

**Results:**

BD-EVs were stratified according to their size (eSEVs and eLEVs) and characterized to define new markers specific to EVs in AD. Both AD-derived eSEVs and eLEVs show the presence of GWAS-associated proteins and indicate a specific AD pathophysiological signature. Notably, AD eSEVs contain more proteins relative to the integrin-mediated synaptic signaling, while AD eLEVs proteins were more related to respiratory electron transport and brain immunity. Injection of these vesicles in transgenic mouse brain revealed that the AD-derived eSEVs are more prone than eLEVs to participate in the prion-like propagation and hence represent an interesting therapeutic target.

**Conclusion:**

This study highlights the significant contribution of AD-derived EVs to tau propagation and provides new insights into different roles of EV sub-populations in AD.

**Supplementary Information:**

The online version contains supplementary material available at 10.1186/s40035-025-00519-z.

## Background

Extracellular vesicles (EVs) are spherical nanoparticles comprised of a bilipid layer. EVs mainly contain proteins, nucleic acids and lipids. However, under this generic name, there is a huge diversity of EVs depending on their biogenesis, cell of origin, content and their surfaceome. This makes EVs a complex spectrum of sub-populations, often classified according to their biogenesis as ectosomes, exosomes and apoptotic bodies [1–4]. Ectosomes, also known as microparticles, are generated by outward budding of the plasma membrane, while exosomes are released by the exocytosis of multivesicular bodies filled with intraluminal vesicles [1]. Classically, exosomes are described as smaller (30–150 nm) than ectosomes (100–500 nm); however, this is a spectrum of secreted EVs that is cell-dependent [3]. Currently, there is a growing interest in EVs in neurodegenerative diseases due to their multiple roles in intercellular communications in both physiological and pathological contexts [5].

Alzheimer’s disease (AD), the most common neurodegenerative disease, has neuro-anatomical features which imply the presence of two hallmark lesions accompanied by an inflammatory reaction during pathology progression [6, 7]. These brain lesions are extracellular amyloid plaques formed by Aβ peptides and intraneuronal neurofibrillary tangles formed by aggregated tau protein. The spatial occurrence of neurofibrillary tangles correlates with the cognitive decline observed in AD patients [8, 9]. A growing body of evidence explains the spreading of tau pathology by a prion-like propagation of pathological tau seeds from an affected cell to a healthy cell [10, 11]. This implicates transfer of tau seeds to the extracellular space before being uptaken by healthy cells. Tau is a cytosolic translated protein and is also detected in the extracellular space such as the cerebrospinal fluid (CSF) of healthy subjects and AD patients [12, 13]. These arguments suggest the existence of unconventional protein secretion mechanisms of tau [14], which can include heparan sulfate proteoglycan-mediated tau translocation, autophagy-mediated secretion or tau encapsulation into EVs [15–19]. Recent lines of evidence, including ours, indicate the presence of pathological tau seeds in brain-derived EVs (BD-EVs) of AD transgenic mouse models [20–22] and AD brain patients [21, 23]. Interestingly, Ruan and collaborators demonstrated that tau within AD BD-EVs is even more seed-competent than free-form tau [23].

Our current interest is now to go further and to understand the involvement of EV subtypes in the complex AD pathophysiology through their protein profiles and their contribution to tau propagation. At this day, more papers can be found on exosomes, which is partially due to a lack of robust EVs nomenclature and to the technical hurdles to distinguish large and small EVs based on their biogenesis [16, 24]. Consequently, there is a lack of comparative studies on EV sub-populations stratified by their biogenesis. Technical challenges in segregating EVs into distinct categories of microvesicles and exosomes lead to increased risk of cross-contamination. This directed our choice for a stratification based on size [3, 4]. Consequently, here, EVs are presently delineated into two main groups: enriched-small EVs (eSEVs, ≤ 150 nm diameter) and enriched-large EVs (eLEVs, > 150 nm diameter). eSEVs and eLEVs from human brain-derived fluid (BDF) were separated by combining size-exclusion chromatography (SEC) and ultracentrifugation (UC). Here, pure and intact EVs were collected from frozen human brain samples after enzymatic dissociation. We aim to define the best enzyme for brain dissociation optimal for downstream proteomic analysis and functional studies. For this, papain (broad substrate specificity: L-Arginine and L-lysine) [25] and collagenase type 3 (specific substrate specificity: triple helix structure of collagen) [26] were compared. Our results revealed an increased protein yield and preservation of transmembrane proteins for both EV sub-populations by using the collagenase enzyme. Further, the proteomic profiling of the collagenase-purified AD eLEVs and eSEVs revealed a unique AD pathology signature where Genome-Wide Association Study (GWAS)-associated proteins were also detected by proteomics. Interestingly, AD-derived eSEVs and eLEVs indicate unique and differentially expressed proteins (DEP), suggesting different implications of EV sub-populations as mediators of dysregulated pathways of AD. This is illustrated here by the assessment of their tau seeding capacity, which demonstrates that AD-derived eSEVs, contrary to AD-derived eLEVs, exhibit an enhanced ability to promote tau spreading in the brain.

## Materials and methods

### Study workflow

Our study aimed to investigate the roles of EV subtypes (eSEVs and eLEVs) in AD pathophysiology. The results are organized as follows:

1. *Validation of EV separation and brain dissociation protocol.* We aimed to define an experimental procedure to recover well-preserved EVs applicable to both control and AD BDF. Due to ethical constraints and the value of human samples, we pooled post-analytical data from AD and controls, except for transmission electronic microscopy (TEM) images, for which a mixed AD/control EVs pool was used.
2. *Identification of AD-related EV phenomena*. To identify common EV-related features in AD, the proteomics analysis was performed on eSEVs and eLEVs from a pool of four AD patients (from collagenase-derived BDF).
3. *Functional assessment of EV seeding capacity*. Using the HEK-tau biosensor cell model and in vivo experiments, we evaluated the seeding capacity of eSEVs and eLEVs from four individual AD or control patients. For in vivo studies, EVs from pooled samples (four AD or four controls) were injected stereotactically to minimize variabilities related to both animals and patients.

A summary of human samples used in each experiment is provided in Table S1.

### Human samples

Non-demented human control (CTRL) and AD prefrontal (Brodmann Area 8/9), fresh-frozen brain extracts were obtained from the Lille Neurobank (fulfilling French legal requirements concerning biological resources and declared to the competent authority under the number DC2008-642) with donor consent, data protection and Ethics Committee approval. Samples were managed by the CRB/CIC1403 Biobank, BB-0033-00030. The demographic data are listed in Table 1. It is important to note that the Lille Neurobank has a limited number of control subjects. Consequently, the control group used in this study spanned a broad age range (22 to 81 years, mean age 56.2 years) and was therefore not age-matched to the Alzheimer’s patient population (mean age 67 years, SD 7.7).

**Table 1:** Demographic, biological and clinical characteristics of the human brain sample donors

Patient ID	Sex	Age of death (years)	PMD (h)	Diagnosis	Tau lesions	Braak stage	Thal stage	Cause of death	*APOE* status
A	F	66	16.5	AD	NFT	VI	4		E3/E3
B	M	78	10	AD	NFT	VI	4		N/A
C	F	60	24	AD	NFT	VI	5		E4/E4
D	M	64	20	AD	NFT	VI	4		E3/E3
E	F	81	N/A	CTRL	none	I	0	Pericarditis	
F	M	22	24	CTRL	none	0	0	Myocarditis	
G	M	59	13	CTRL	none	0	0	Septic shock	
H	M	41	11	CTRL	none	0	0	Suffocation	
I	M	78	19	CTRL	none	0	0	Invasive aspergillosis	

The ages of control F and H were 22 and 41 years, respectively, which were much younger than the average of AD patients (67 years). PMD post-mortem-delay, NFT neurofibrillary tangles. N/A not available

### Brain-derived fluid isolation

Purification of BD-EVs is a challenge since the preparation of BDF was done from frozen human prefrontal brain extracts. To isolate the BDF from this solid tissue, enzymatic dissociation was used. Here, two different protocols were applied. The first protocol used papain for enzymatic digestion of the brain tissue as previously described [20] and adapted in our previous study [21]. Briefly, brain tissue (80 mg for TEM; 400 mg for nanoparticle tracking analysis [NTA] and proteomic analysis) was incubated on ice in Hibernate-A and then gently homogenized in a Potter before adding 2 mL of 20 units/mL papain (LS003119, Worthington Biochemical Corporation, Lakewood, NJ). After a 20 min incubation at 37 °C with agitation, 15 mL of cold Hibernate-A (50 mmol/L NaF, 200 nmol/L Na_3_VO_4_, 10 nmol/L protease inhibitor (E3132, Sigma, St. Quentin Fallavier, France) and protease inhibitor cocktail (04693116001, Roche, Basel, Switzerland)) were added and mixed by pipetting to stop the enzymatic activity while on ice.

The second protocol used collagenase type 3 (LS0004182, Pan Biotech, Aidenbach, Germany) for enzymatic tissue dissociation as previously described by Vella and collaborators [27]. Briefly, brain tissue (80 mg for TEM; 200 mg for NTA and proteomic analysis) was sliced on ice to generate smaller sections (~ 2 mm) before adding 75 units/mL of collagenase type 3 in Gibco™ Hibernate™-E Medium (800 μL per 100 mg of tissue, 10315538, Thermo Fisher Scientific, Waltham, MA). After 20 min incubation at 37 °C with agitation, PhosSTOP (4906837001, Roche) and Complete Protease Inhibitor including EDTA (4693124001, Roche) were added to a final concentration 1× on ice.

For both protocols, successive centrifugations of 300 × *g*, 2000 × *g* and 10,000 × *g* were applied at 4 °C to remove cells, membranes and debris, respectively. The final supernatant was entitled BDF and was consistently prepared freshly prior to each EV isolation.

### BD-EV isolation

First, SEC was used to isolate EVs from the BDF and to separate EVs from proteins contaminants [28, 29]. SEC allows quick isolation with little non-vesicular contaminants. Commercial SEC columns (IC0-70, IZON) packed with Sepharose resin CL-2B (CL2B300, Sigma-Aldrich) were used. After column equilibration with degassed phosphate-buffered saline (PBS, 12559069, Gibco), 500 µL of BDF were applied on the SEC column followed by elution in degassed PBS. After the void volume (3 mL), the first 2 mL (F1-4) were recovered as EVs fraction in protein low binding tubes (0030108132, Eppendorf protein LoBind, Hamburg, Germany). We previously characterized this fraction enriched with BD-EVs [21] in accordance to the MISEV 2018 guidelines [3].

### Separation of large from small BD-EVs

SEC is not able to fully separate EV subtypes depending on their size. This results in a mix of both large- and small-sized EV subtypes in the SEC-derived EV fraction (2 mL). Hence, a centrifugation step at 10,000 × *g* for 30 min at 4 °C was added to pellet eLEVs (Centrifuge 5424-R, 2519550, Eppendorf). The supernatant was transferred to an Ultra-Clear™ Centrifuge Tubes (344062, Beckman Coulter Inc., Brea, CA) and the pellet was suspended in 500 µL of ice-cold PBS. This 10,000 × *g* centrifugation step was repeated two more times to reduce contamination of the eLEVs pellet by eSEVs from the supernatant. The final pellet representing the eLEVs fraction was suspended in 50 µL PBS. At the end, the supernatant (3 mL) from all three centrifugations was ultracentrifuged at 20,000 × *g* for 2 h at 4 °C (Optima XE-90, rotor SW60Ti, Ultra-Clear) to pellet residual eLEVs and recovered only eSEVs in the final supernatant. This supernatant enriched with eSEVs was concentrated using ultrafiltration device Amicon Ultra-2 3 kDa (UFC200324, Millipore) at 4000 × *g* (Multifuge X3R, Thermo Scientific, Waltham, MA) to a final volume of 100 µL.

### NTA

The concentration and size distribution of particles were measured by NTA (NanoSight NS300, Malvern Panalytical, Malvern, UK) immediately after isolation. eSEVs and eLEVs from two CTRL and one AD patient were passed separately on the NTA and were pooled post-analysis (Table S1). Samples were diluted in PBS and continuously infused into the NTA device by an automatic syringe pump at a flow rate of 20 μL/min. The focus was adjusted and the temperature was set to 25 °C. Three videos of 60 s were acquired at camera level 15 and processed at detection level 4 using the NTA software (v 3.2.16). Samples were freshly used for TEM or stored at -20 °C until proteomic analysis.

### TEM

TEM morphological visualization of eLEV and eSEV fractions was performed using BDF of a pool of two controls and one AD patient (Table S1). For this pool, 80 mg brain tissue resulted in approximately 600 µL BDF, of which 500 µL was used for one SEC and downstream eSEVs and eLEVs separation. Then, 5 µL of eLEVs or 5 µL eSEVs sample was deposited on a carbon grid (400 mesh) and incubated for 20 min at room temperature (RT). Grids were rinsed twice in PBS and were fixed in PBS-glutaraldehyde (1%) for 5 min at RT and then rinsed 7 times in distilled water. The light-sensitive grids were incubated for 5 min in 1% uranyl acetate and for 10 min on ice in a mixture containing 4% uranyl acetate/2% methylcellulose (25 cP, 9004-67-5, Sigma) in the dark. Dry grids were observed under a transmission electron microscope (Zeiss EM900) with a 20,000 × objective.

### Proteomic sample preparation

eLEVs and eSEVs were isolated from either a pool of four CTRL or a pool of four AD patients (Table S1). These were prepared with both brain-dissociation protocols, namely papain or collagenase dissociation and were further analyzed by label free quantification mass spectrometry at the OrganOmics platform of PRISM Inserm U1192 (Lille, France). Using both dissociation enzymes, around 400 mg of brain tissue were used, resulting in 3 mL of BDF, which was suited for six SEC. More precisely, same numbers of eLEVs and eSEVs (3.2 × 10^9^) were lysed in RIPA buffer for 15 min at 95 °C and subsequently centrifuged at 16,000 × *g* for 10 min. For each sample, the collected supernatant was reduced using reduction buffer (Dithiothreitol, DTT 0.1 mol/L) for 40 min at 56 °C, and diluted in the denaturing buffer (0.1 mol/L Tris/HCl, 8 mol/L urea, pH 8.5). The preparation of samples by FASP (Filter Aided Sample Preparation) [30, 31] was carried out using 30 kDa Amicon device (UFC503024, Millipore, Burlington, MA) to eliminate the denaturant buffer by centrifugation at 14,000 × *g* for 15 min. This rinsing step was repeated a second time. Next, alkylation of the proteins was done by addition of IAA buffer (Iodoacetamide 0.05 mol/L) in the Amicon and set for 20 min in the dark. This was followed by a centrifugation at 14,000 × *g* for 10 min. Samples were washed three times by addition of denaturing buffer and followed three times by addition of AB buffer (ammonium bicarbonate 0.05 mol/L). At each washing step, centrifugation was carried out at 14,000 × *g* for 10 min. Samples were then incubated by adding 40 µL of trypsin buffer (40 µg/µL, in AB buffer) at 37 °C overnight. The digested proteins were collected by centrifugation at 14,000 × *g* for 10 min and rinsed on Amicon device with 0.5 mol/L NaCl. The digestion was stopped using 5% trifluoroacetic acid. The samples were desalted using Evotips-C18 (EV2018, Evosep, Odense, Denmark) in accordance with the manufacturer's instructions provided by Evosep, immediately before data acquisition via mass spectrometry (MS).

### Liquid chromatography coupled to tandem mass spectrometry (LC–MS/MS) analysis

The Evotips, housing the peptides, were introduced into the Evosep-One [32] liquid chromatography system (Evosep, Odense, Denmark). The system automatically engaged the tip and conducted elution directly within the liquid chromatography setup. Peptide separation occurred utilizing the C18 endurance column (15 cm × 150 μm ID, 1.9 μm) employing the extended method 15 SPD (Sample Per Day). Mobile phase A consisted of 0.1% formic acid (FA) in water, while phase B comprised 0.1% FA in acetonitrile. The chromatographic system was linked to a Q-Exactive Orbitrap mass spectrometer (Thermo Scientific) through a nanospray source. The Q-Exactive operated in a data-dependent acquisition mode, targeting the top 10 most intense ions for MS analysis. MS analysis spanned a mass to charge range (m/z) of 300 to 1600, with a resolution of 70,000 full width at half maximum, an automated gate control of 3 × 10^6^ ions and a maximum injection time of 120 milisecondes (ms). For MS/MS analysis, the m/z range extended from 200 to 2000, with an AGC of 5 × 10^4^ ions, a maximum injection time of 60 ms, and a resolution set at 17,500 FWHM. Higher Energy Collision Dissociation was set to 30%, with precursor ions bearing charge states >  + 1 and <  + 8 selected for fragmentation, and a dynamic exclusion time of 20 s. The mass spectrometry proteomics data have been deposited to the ProteomeXchange Consortium via the PRIDE [33] partner repository with the dataset identifier PXD055734. List of proteins and differentially expressed proteins are also listed in Tables S2 and S3.

### Proteomic data analysis

Proteins were identified through comparison of all MS/MS data with the *Homo sapiens* proteome database (Uniprot, release August 2022, 75,004 entries) using the MaxQuant software (version 1.6.0.5) [34, 35]. An initial mass tolerance of 6 ppm was applied for MS mode, while a tolerance of 20 ppm was set for fragmentation data in MS/MS mode. Digestion parameters utilized trypsin with up to 2 allowed missed cleavages. Variable modifications included oxidation of methionine and N-terminal protein acetylation, while carbamidomethylation of cysteine was selected as a fixed modification. Label-free quantification (LFQ) was conducted with default parameters of the MaxLFQ algorithm [36]. Protein and peptide identification adhered to a false discovery rate of 1%, with a requirement of at least 2 peptides per protein, including 1 unique peptide. Statistical analysis was performed using Perseus software (version 1.6.10.43). Briefly, LFQ intensities for each sample were imported into Perseus after which the data matrix underwent filtering to remove potential contaminants, reverse entries and those identified by site only. Data transformation involved log2(x) conversion. Prior to statistical analysis, groups were defined with 3 replicates per group. Assessment of DEP between two groups was done using a Student's *t*-test with a significance threshold of *P*-value set to 0.01. To visualize these proteins, a volcano plot was generated with an artificial within-group variance (s0) of ± 0.1 and a –log (*P*-value 0.05) of 1.3.

The EV quality was assessed using our proteomic data. For this, our data were crossed with the 5 categories of the Minimal Information for Study of Extracellular Vesicles guidelines 2023 (MISEV2023) [4] which were complemented with the protein lists of the MISEV guidelines 2018 (MISEV2018) [3]. Categories 1 and 2 are defined as EV-enriched proteins while category 3 was defined as EV contaminants. The complete list of detected and non-detected proteins for each category is shown in Table S4. To analyze the benefit of collagenase brain dissociation, a fold enrichment was calculated. For this, the number of present proteins in the collagenase data (eSEVs and eLEVs) over the number of proteins present in the papain database (eSEVs and eLEVs) was analyzed for MISEV2023 categories 1 to 3.

The assessment of transmembrane proteins was done by crossing our identified proteins (Uniprot entry) with the UniProtKB reviewed (Swiss-Prot) human transmembrane protein database (5232 proteins, KW-0812). The enrichment analysis for the gene ontology “biological processes” (GOBP) and “cellular components” (GOCC) categories was performed with the Funrich software (version 3.1.4). For GOCC the detected exosomes and lysosomes categories had around 64% of protein overlap due to common pathways between intraluminal vesicles and secretory vesicles. Further, only 21% were lysosome-specific proteins.

### Western blot

For western blotting, eSEVs and eLEVs were obtained from a pool of four CTRL or a pool of four AD patients (Table S1) following the protocol described above. For western blot, 125 mg of brain tissue, resulting in 1 mL BDF, allowed two SEC and further size separation. The eSEVs were additionally concentrated by an ultracentrifugation step at 100,000 × *g* for 2 h at 4 °C. The pellet of eSEVs was suspended in PBS (25 µL PBS for each SEC). Concentrations of both eSEVs and eLEVs were quantified by NTA. 5 × 10^9^ EVs were diluted in RIPA 1× (150 mmol/L NaCl, 0.1% SDS, 0.5% Sodium deoxycholate, 1% NP-40, EDTA free protease inhibitor, 50 mmol/L Tris base at pH 8) and sonicated in a water bath (Bioruptor sonication system, Diagenode, Liège, Belgium) for 5 min on high intensity setting. The EVs were then diluted in lithium dodecyl sulphate (LDS 2×: NuPage LDS sample buffer 2×, NuPage reducing agent 2×) and heated for 10 min at 100 °C. After this, eSEVs and eLEVs were loaded on 4%–12% Bis–Tris NuPAGE Novex gels (NP0336PK2, Invitrogen, Waltham, MA) and transferred to a nitrocellulose membrane of 0.45 µm employing the Novex system from Life Technologies (XCell II blot module, Carlsbad, CA). Membranes were blocked in Tris-buffered saline with 5% skim milk for 1 h at RT and incubated with the appropriate primary antibody overnight at 4 °C in TNT buffer (30 mM Tris, 280 mM NaCl, 0.1% Tween20) complemented with 5% milk. Antibodies are listed in Table 2. Membranes were then rinsed and further incubated with horseradish peroxidase-labelled secondary antibodies and bands were visualized by chemiluminescence (ECL, Amersham Biosciences, Little Chalfont, Buckinghamshire, UK).

**Table 2:** List of primary and secondary antibodies used for western blotting of eLEVs and eSEVs

Primary antibody	Manufacturer, reference	Dilution	Secondary antibody	Supplier	Dilution
FERMT2	GeneTex, GTX84507	1/1000	Horse anti-mouse IgG (H + L) peroxidase	Vector Laboratories, PI-2000	1/50,000
Clusterin (CLU)	Abcam, ab92548	1/1000	Goat anti-rabbit IgG (H + L) peroxidase	Vector Laboratories, PI-1000	1/5000

### Cell culture

The stable Tau RD-P301S FRET Biosensor cells (CRL-3275, ATCC, Manassas, VA) and HEK 293 T cells (CRL-3216, ATCC) were cultured in Dulbecco’s modified Eagle’s medium (DMEM with pyruvate and without HEPES, 13345364, Gibco, Grand Island, NY) complemented with 10% fetal bovine serum (FBS, A5256701, Gibco), glutaMax 1× (35050061, Gibco) and 1% penicillin–streptomycin. Cells were maintained in a humidified incubator with 5% CO_2_. The cells were split twice a week.

### Fluorescence resonance energy transfer (FRET) biosensor cell assay

Upon addition of a tau seed, the tau-RD with a P301S pro-aggregative mutation coupled with a Yellow-fluorescent or Cyan-fluorescent reporter come in close proximity and allow a FRET measurement of these HEK-tau FRET cells. To assess the in vitro seeding capacity of AD eSEVs and eLEVs, the FRET biosensor cell assay was performed as previously described in Leroux and collaborators [21]. Briefly, HEK-tau FRET and HEK 293 T cells were plated (150,000 cells per well) 24 h before lipofection of 50 µL of eLEVs or eSEVs from individual AD patients or individual CTRL (Table S1). Seventy-two hours after lipofection, cells were analysed on the Aria SORP flow cytometer (BD Biosciences; acquisition software FACSDiva v7.0, BD Biosciences, San Jose, CA). The FRET data were quantified using the Kaluza analysis software v2 and results were expressed as the percentage of FRET-positive cells. eSEVs and eLEVs from four individual AD patients or four CTRL were evaluated in duplicate. Three independent experiments were performed. At least 10,000 cells per replicate were analyzed.

### Animals

The study was performed in accordance with French and European Community rules. The experimental research was performed with the approval of the local animal ethics committee (Comité d’éthique en expérimentation animale, CEEA) under APAFIS agreement (#43474-2023050714441306 v6) and follows European guidelines for the use of animals. The animals (males and females) were housed in a temperature-controlled room (20–22 °C) and maintained on a 12-h day/12-h night cycle with food and water provided ad libitum in a specific, pathogen-free animal facility (with five mice per cage). Animals were randomly allocated to the different experimental groups. The tau transgenic mouse line THY-Tau30 expressing human 1N4R tau protein with two pathogenic mutations (P301S and G272V) under the control of the neuron-specific Thy-1.2 promoter was used [37].

### Stereotaxic injections

To assess the in vivo seeding capacity of eSEVs and eLEVs, stereotaxic injections were performed in THY-Tau30 mice as described in Leroux and collaborators [21]. Briefly, around 350 mg brain tissue from a pool of four CTRL or four AD patients (Table S1) were dissociated using collagenase enzyme. The obtained 2.5 mL BDF allowed 5 SEC and downstream eSEVs and eLEVs separation through differential centrifugations. The eSEVs and eLEVs were maximally concentrated using 3 K Amicon. For each condition, 2.5 µL (1.6 × 10^9^ vesicles) were bilaterally injected into the hippocampi of 1-month-old anesthetized (ketamine (100 mg/kg) and xylazine (20 mg/kg)) THY-Tau30 mice (*n* = 4 for control eSEVs and *n* = 5 mice for CTRL eLEVs, AD eSEVs and AD eLEVs) at a flow rate of 0.25 mL/min followed by a 5 min syringe hold. Injection coordinates were anterior–posterior, − 2.5 mm; mediallateral, − 1 mm; dorsal–ventral, − 1.8 mm to bregma [38]. In contrary to the FRET assay, no lipofectamine was used for the stereotactic injected material.

### Tissue processing and immunohistochemistry (IHC)

Brain tissue processing and IHC were performed as explained in Leroux and collaborators [21]. Briefly, at four weeks after stereotaxic injection, mice underwent trans-cardiac perfusion with 0.9% saline solution followed by 4% PFA perfusion. Extracted brains were post-fixed before isopentane freezing. Using the cryostat microtome, free-floating coronal sections (40 µm thickness) were obtained. For IHC, the brain sections were washed, treated with 0.3% H_2_O_2_, rinsed and Mouse on Mouse blocking reagent (MKB-2213-1, Vector Laboratories, Newark, DE) was added. After three rinses, overnight incubation with the primary antibody MC1-biotin recognising the pathological tau conformation [39] (generously donated by Dr. Peter Davies, Feinstein Institute for Medical Research, Manhasset, NY, USA) was done, followed by rinses and amplification using anti-mouse biotinylated IgG (BA-9200-1.5, Vector Laboratories) and application of the avidin–biotin-HRP complex (PK-6100, Vector Laboratories). Visualisation of tau lesions was done using diaminobenzidine tetrahydrochloride. Next, brain sections were mounted, air-dried and dehydrated by passage through a graded series of alcohol and toluene baths. Lastly, coverslips were mounted with VectaMount and images were acquired using a Zeiss Axio Scan.Z1 and scale bar were added using the ZEN Blue software (version ZEN 2.3 lite).

### Tau lesion quantification

Mounted brain sections were visualized on the Mercator Leica DM5500 where a threshold of MC1-positive lesions was established manually to present a minimum background and remained constant throughout the analysis. For blinded quantification of MC1 immunoreactivity, the CA1 region of the hippocampus from bregma − 1.06 to bregma − 3.52 (based on the Mouse Atlas, George Paxinos and Keith B.J. Franklin, Second Edition, Academic Press) was chosen as the quantification zone. The number of MC1-positive somas were blindly and manually counted per brain section by two independent individuals. Results are presented as the mean number of neurofibrillary tangles per brain section where the left and right hemispheres of the mice were counted separately.

### Statistical analyses

Statistics and graphs were generated using the GraphPad Prism 9 software (version 9.1.0). Data are presented as mean ± SEM. Shapiro–Wilk normality test was used to assess normality for each group. For comparison of two independent groups with a normal distribution, a *t*-test was done and for groups with non-parametric distributions, a Mann–Whitney U test was used. Comparisons of three or more independent groups with a normal distribution were performed using ordinary one-way ANOVA, while Kruskal–Wallis test was used for non-parametric samples. Statistical testing was done at the two-tailed *P*-value of 0.05.

## Results

### Isolation of high-quality small and large human BD-EVs

The high heterogeneity of EVs based on their biogenesis and the absence of biogenesis-specific EV biomarkers, have prompted the development of a size-based segregation protocol for BD-EVs. This was achieved through a combination of SEC followed by UC (Figs. S1a and 1a). This protocol was applied to prefrontal BDF of AD patients and non-demented controls (demographic specifications presented in Table 1) and showed no significant loss in EV quantity (Fig. S2).

**Fig. 1:**
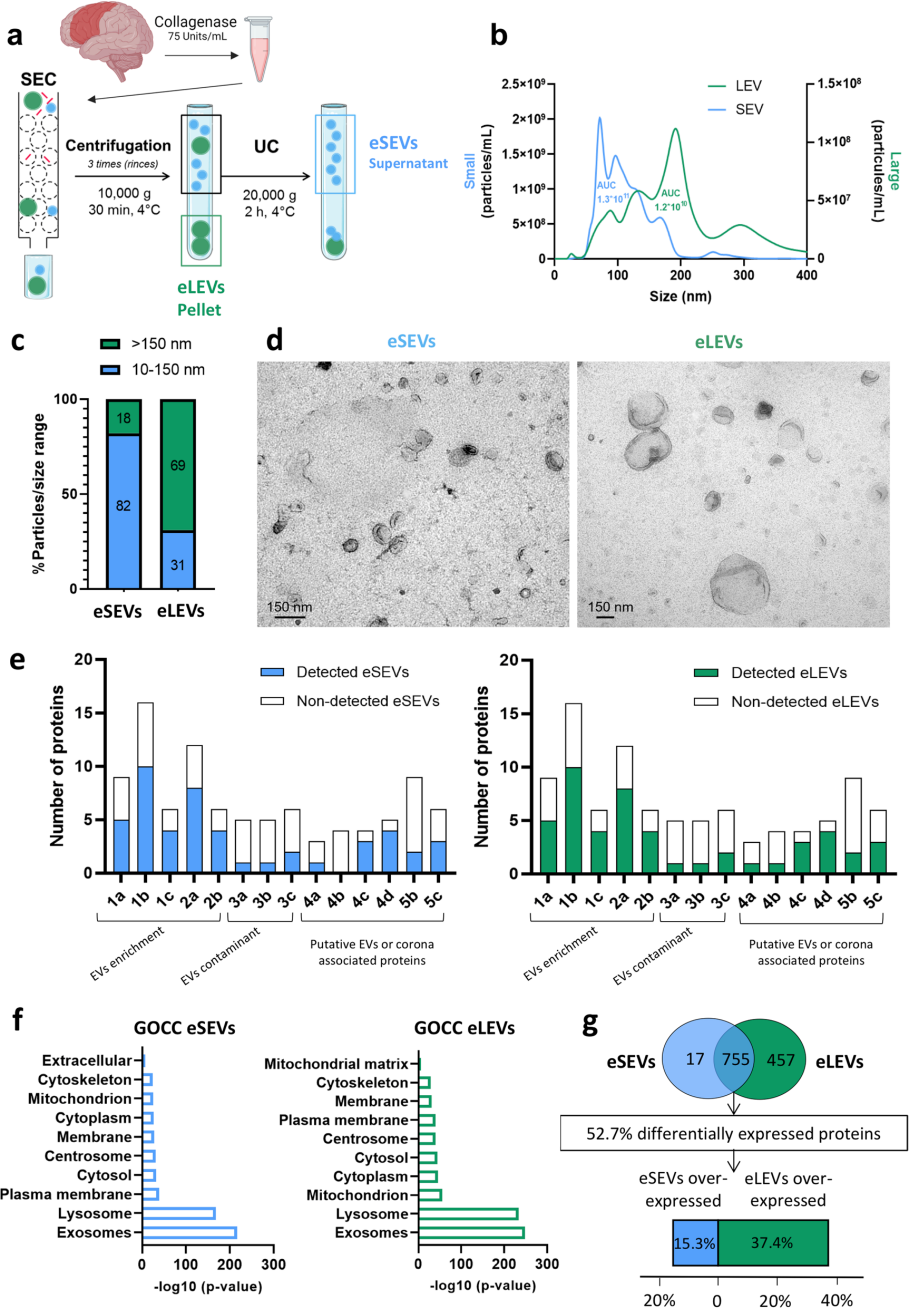
New size-based EV separation protocol of BDF using collagenase type 3 dissociation.** a** BDF was obtained by collagenase type 3 enzymatic dissociation following the protocol as described by Vella and collaborators [27] for AD and CTRL brain extracts. **b** Size distribution of eSEVs and eLEVs was determined by nanoparticle tracking analysis (NTA). Data are presented as the mean of 2 individual CTRL and one AD.** c** eSEVs and eLEVs separation enrichment was calculated from NTA and indicated an 82% size enrichment for eSEVs and 69% for eLEVs. **d** Morphology of eSEVs and eLEVs visualized by TEM. Scale bars, 150 nm. Images are coming from a pool of 2 CTRL- and 1 AD-derived BD-EVs.** e** Number of proteins detected in eSEVs (left) and eLEVs (right) as recommended by the MISEV2023 guidelines after mass spectrometry-based proteomic analysis [3]. MISEV2023 categories; 1a: Multi-pass TM proteins associated with plasma membrane and/or endosomes; 1b: Single-pass TM proteins associated with plasma membrane and/or endosomes; 1c: GPI- or lipid-anchored proteins associated with plasma membrane and/or endosomes; 2a: Cytosolic proteins with lipid or membrane protein-binding ability; 2b: Cytosolic proteins with promiscuous incorporation into EVs; 3a: Lipoproteins; 3b: Protein and protein/nucleic acid aggregates; 3c: Exomere or supermere-enriched components; 4a: Nucleus; 4b: Mitochondria; 4c: Secretory pathway: Endoplasmic reticulum, Golgi apparatus; 4d: Autophagosomes, cytoskeleton; 5b: Cytokines and growth factors; 5c: Adhesion and extracellular matrix proteins. **f** Horizontal barplot of –log (*P*-value) from LFQ intensities obtained for 10 selected GOCC terms after quantitative proteomic analysis of eSEVs (left) and eLEVs (right). **g** Venn diagram (top) indicating unique proteins for both eSEVs and eLEVs and 755 common proteins of which 52.7% were found differentially expressed. The boxplot represents the repartition of differentially expressed proteins between eSEVs and eLEVs (bottom). For **e** to **g**, EVs are coming from a pool of 4 CTRL and from a pool of 4 AD (post-mass spectrometry fusion). For **a** to **g**, eSEVs are represented in blue and eLEVs in green

**Fig. 2:**
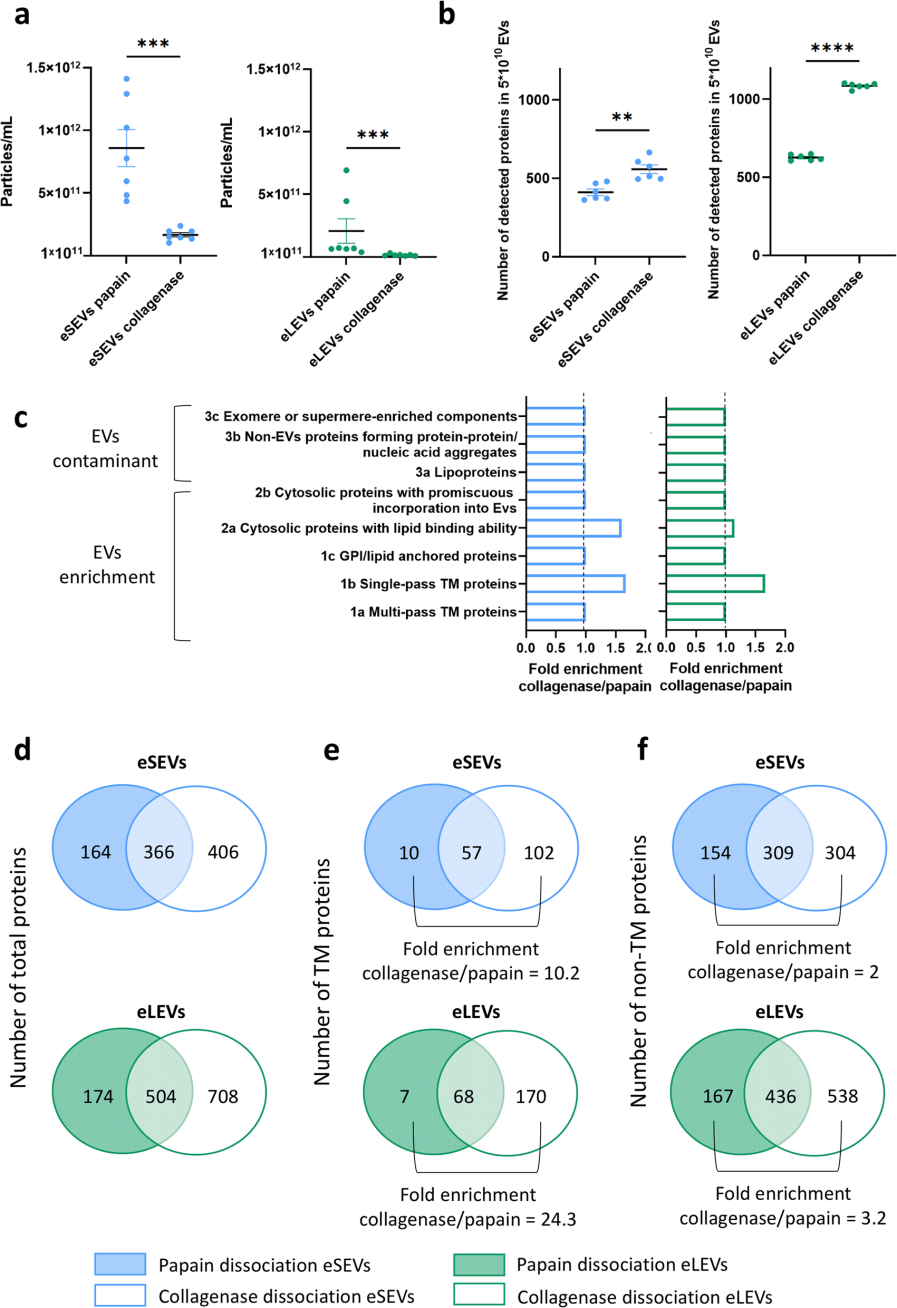
Comparison of enzymatic brain dissociation protocols for downstream analysis of eSEVs and eLEVs. **a** Nanoparticle tracking analysis measurements of concentrations of eSEVs and eLEVs from papain and collagenase enzymatic brain dissociation from individual patients (4 CTRL and 3 AD). For eSEVs, unpaired *t*-test was used for parametric comparison between papain and collagenase. For eLEVs, Mann–Whitney test was used for non-parametric comparison between papain and collagenase. ***P < 0.001. **b** Dotplots comparing the number of proteins detected by mass spectrometry for 3.2 × 10^9^ EVs (3 technical replicates of BD-EVs from a pool of CTRL plus 3 technical replicates of BD-EVs from a pool of AD). For eSEVs and eLEVs, unpaired *t*-test was used for parametric comparison between papain and collagenase. ** P < 0.01, **** P < 0.0001. Collagenase allows a better downstream protein identification. **c** Horizontal barplots indicating the protein fold enrichment of collagenase compared to papain according to the MISEV2023 guidelines. This enrichment revealed a higher presence of single-pass transmembrane (TM) proteins and proteins known to be detected in EVs, while maintaining equal contaminant levels. This indicates a higher quality of both eSEVs and eLEVs using collagenase 3 as dissociation enzyme. **d-f** Venn diagrams to compare the number of total proteins (**d**), TM proteins (**e**) and non-TM proteins (**f**) identified by mass spectrometry for eSEVs and eLEVs purified from papain (shaded) and collagenase (transparent). This quantitative protein comparison indicated that more TM and non-TM proteins can be identified by mass spectrometry using collagenase brain dissociation. For **c** to **f**, EVs were from a pool of 4 CTRL and from a pool of 4 AD. For **a** to **f**, eSEVs are represented in blue and eLEVs in green

Two enzymatic dissociation protocols, namely papain and collagenase type 3, were compared to assess their impact on BD-EV size, yield, purity and integrity (Fig. S1b, Figs. 1b and 2, Tables S2-S4. Both enzymatic protocols successfully enriched the two size-based EV populations: eSEVs (10–150 nm) and eLEVs (> 150 nm), which was confirmed by NTA (Fig. S1b and Fig. 1b). Papain-derived eSEVs and eLEVs contained 73% and 77% of vesicles in their respective size ranges (Fig. S1c), and collagenase-derived fractions contained 82% and 69% of respective size range enrichment (Fig. 1c). TEM confirmed intact EV morphology with preservation of the cup-shape of EVs (Fig. S1d and Fig. 1d).

The proteomic analyses validated by MISEV guidelines confirmed the presence of EV-enriched proteins (categories 1 and 2) with minimal contaminants (category 3) (Fig. S1e and Fig. 1e; Table S4) [3, 4]. Proteins from categories 4c and d were also identified in both eSEVs and eLEVs. The presence of these proteins is associated with pathways involved in EV genesis, such as the secretory pathway (including the endoplasmic reticulum and Golgi apparatus) and autophagosomes. Therefore, their presence in certain EV subtypes is not considered as contaminant [4].

GOCC analysis indicated a highest enrichment in exosomes GOCC for both eSEVs and eLEVs (Figs. 1f and S1f). Unique and common protein profiles between eSEVs and eLEVs revealed a higher number of unique proteins in eLEVs compared to eSEVs for both papain (178 vs 30) and collagenase (457 vs 17). More than 50% of common proteins between eSEVs and eLEVs were found differentially expressed (Figs. 1g and S1g). The principal component analysis (PCA) (Fig. S3a, b), as well as the enriched proteins for eSEVs and eLEVs (Fig. S3c, d), confirmed separation of distinct EV sub-populations using our SEC-UC protocol.

Comparison of the brain dissociation enzymes revealed that papain dissociation yielded a significantly higher number of EVs, yet collagenase-derived EVs showed superior downstream proteomic detection by MS (Fig. 2a, b).

A fold enrichment of the number of proteins detected within the MISEV guideline categories was performed for collagenase EVs over papain EVs (Fig. 2c) [4]. This indicated an enrichment in collagenase eSEVs and eLEVs for EV-specific proteins (MISEV categories 1b and 2a) with low presence of contaminants (MISEV category 3). Next, a comparison of transmembrane (TM), luminal (non-TM) and total proteins unveiled more unique proteins in collagenase-derived EVs compared to papain-derived EVs (Fig. 2d–f). The collagenase-derived eSEVs and eLEVs demonstrated a 10.2- and 24.3-fold enrichment in TM proteins over papain-derived fractions, respectively (Fig. 2e). Some TM proteins defined as classical EVs biomarkers, such as CD81 and CD9, were detected using both dissociation protocols, while others like TSG101 and CD63 were absent in both collagenase- and papain-derived EVs. Importantly, EV-associated proteins like CAV1 (Caveolin-1), CAV2 (Caveolin-2), Flotillin-1 (FLOT1), Integrin alpha-1 (ITGA) and ROCK (Rho-associated protein kinase 2) were lost after papain enzymatic digestion (Fig. S4, Table S8) [1]. As some of these TM proteins are crucial for the improved isolation and categorization of EV sub-populations, this also points toward well-preserved EVs after collagenase brain dissociation.

Overall, the combined SEC-UC protocol effectively enriched size-specific EV sub-populations for both papain and collagenase dissociation. However, collagenase dissociation demonstrated enhanced EV purity (enrichment of EV-specific proteins) and integrity (higher TM and non-TM protein detection). Additionally, using collagenase, we isolated 518 proteins that are common to the 948 proteins (54.7%) isolated in the Muraoka study from papain BD-EVs (whole EVs without size stratification) [40] that used higher-powered, non-pooled replicate analysis (Table S9).

Consequently, collagenase was selected as the optimal dissociation enzyme for investigating the role of BD-EV sub-populations in AD pathophysiology.

### GWAS-associated proteins found inside eSEVs and eLEVs from AD patients reveal pathways dysregulated in AD patients

**Fig. 3:**
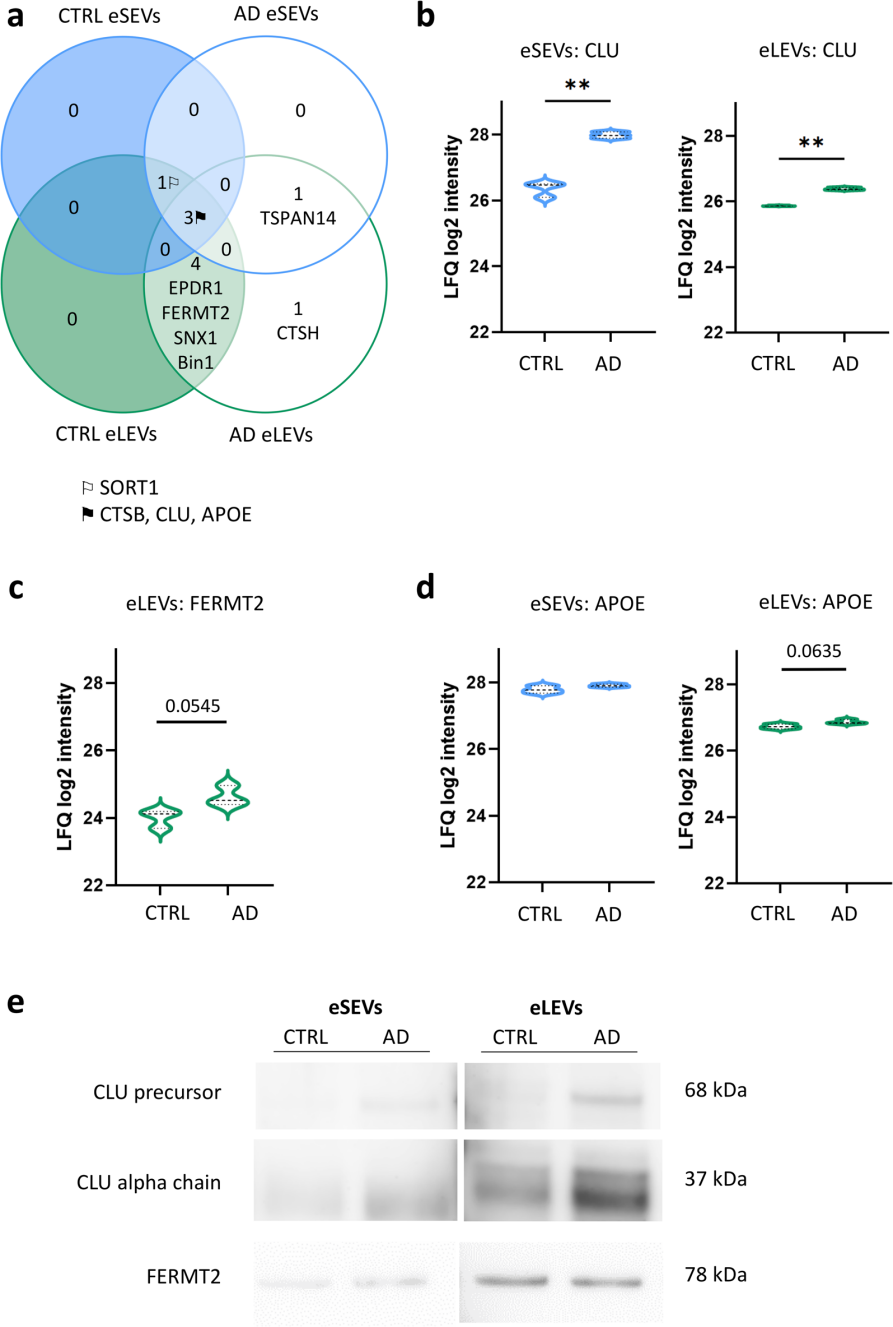
Differential expression profiles of GWAS genes in AD collagenase-derived eSEVs and eLEVs. Stratified BD-EVs came from a pool of 4 CTRL or a pool of 4 AD. **a** Venn diagram representing the GWAS-associated proteins found in eSEVs and eLEVs. This analysis was done by crossing our protein data of AD and CTRL eLEVs and eSEVs with the protein list of GWAS genes [41]. **b-d** Statistical analysis represented in violin plots to compare a few of the GWAS-associated proteins: CLU (**b**), FERMT2 (**c**) and APOE (**d**) identified inside eLEVs and eSEVs between AD and CTRL. For CLU, Mann–Whitney for non-parametric test comparing AD to CTRL. For FERMT2 and APOE, unpaired *t*-test for parametric test comparing AD to CTRL. *P*-values were calculated on LFQ log2 intensities. **P < 0.01. **e** Western blots of CLU and FERMT2 in eSEVs and eLEVs of CTRL and AD. For **a** to **d**, eSEVs are represented in blue and eLEVs in green

AD is a complex progressive disease overruled by sporadic cases compared to inherited cases. A growing field aims to define risk genes, which interlink AD patients compared to controls. This is done in GWAS where peculiar single nucleotide polymorphisms (SNPs) are compared between controls and AD patients [41]. These GWAS studies have already revealed numerous SNPs from genes with dysregulated pathways in AD. Our goal was to assess with an unbiased approach if some proteins found in eSEVs and eLEVs of AD patients are coming from genes identified by GWAS, and thus might participate in the pathological role of EVs in AD. This was done by crossing our collagenase protein database with the GWAS gene list [41]. This analysis did show the presence of GWAS-associated proteins in both eSEVs and eLEVs from AD and CTRL (Fig. 3a). Some GWAS-associated proteins such as Myc box-dependent-interacting protein 1 (BIN1), EPDR1 (mammalian ependymin-related protein 1), fermitin family homolog 2 (FERMT2) and SNX1 (sorting nexin-1) were only detected in eLEVs. Tetraspanin-14 (TSPAN14) and cathepsin H (CTSH) were found only within AD EVs (Fig. 3a, Table S5). Interestingly, we showed a significant increase of LFQ values for the presence of clusterin (CLU) in AD eLEVs and eSEVs compared to CTRL (Fig. 3b). Western blots loaded with the same number of EVs (5 × 10^9^ EVs) allowed comparison of GWAS-associated proteins in eSEVs and eLEVs between AD and CTRL (Fig. 3e and Fig. S5). An increased presence was clearly visualized for both the CLU precursor and cleaved protein in AD patients. Further, a strong tendency of increase of FERMT2 protein LFQ intensities in AD eLEVs compared to CTRL eLEVs was observed (*P* = 0.054) (Fig. 3c). Western blots confirmed the presence of FERMT2 in human eLEVs and eSEVs, but did not show a clear increase in AD eLEVs (Fig. 3e and Fig. S5a). In the brain parenchyma, a strong upward tendency (*P* = 0.057) was noticed for CLU in AD, but no significant difference in FERMT2 level was quantified in AD brains compared to CTRL (Fig. S5b, c).

Taken together, our data unveiled the presence of GWAS-associated proteins in eSEVs and eLEVs. As GWAS-associated proteins are implicated in numerous dysregulated pathways in AD, we seek to identify the biological pathways reflected within AD-derived EV sub-populations.

### Biological pathway profiling of eSEVs and eLEVs reveals a specific AD signature

**Fig. 4:**
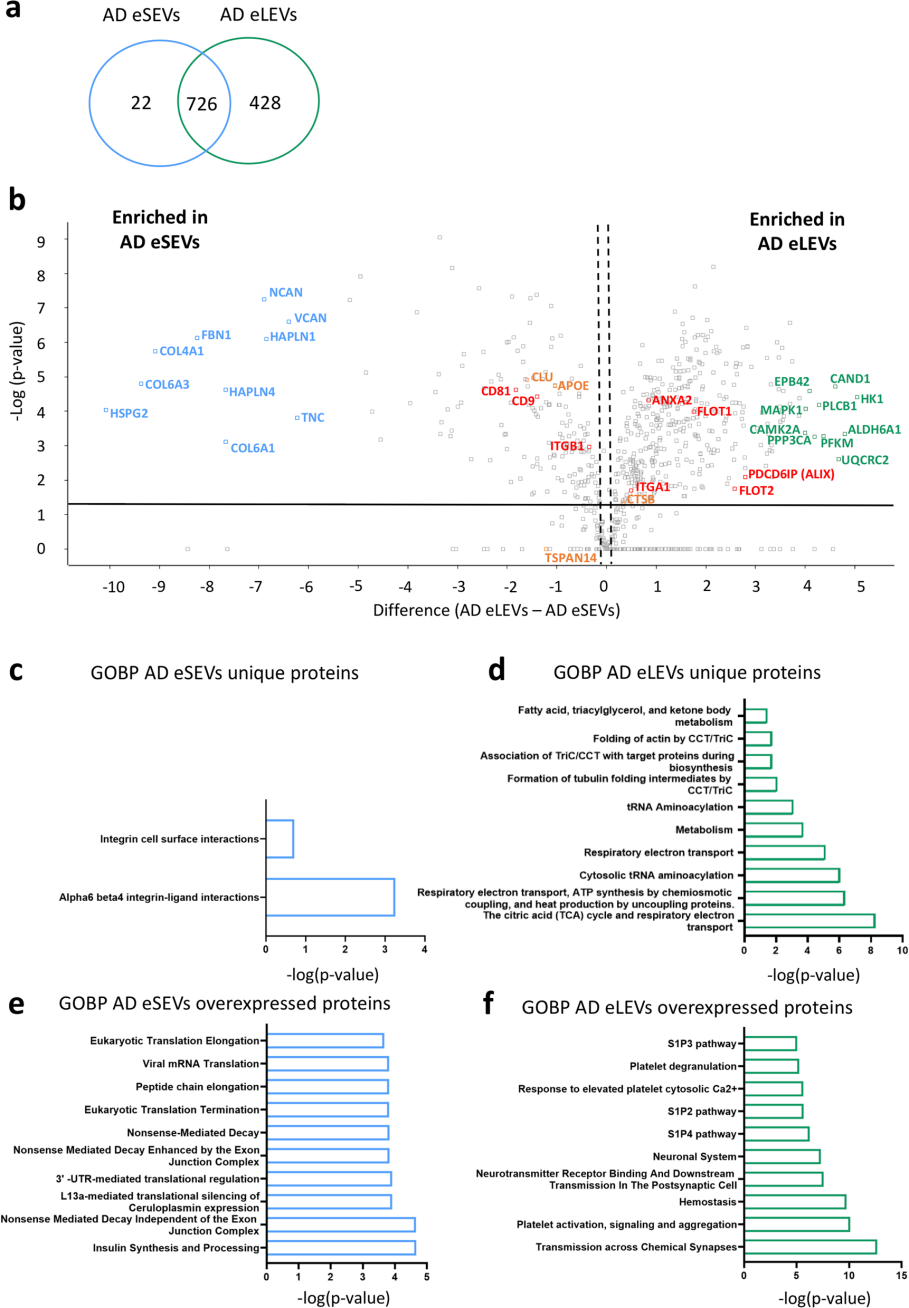
Proteomic profiling of AD-derived eSEVs and eLEVs. **a** Venn diagram indicating the number of unique and shared proteins between AD eSEVs and AD eLEVs. This indicated 22 and 428 unique proteins respectively for AD eSEVs and AD eLEVs. **b** Volcano plot of quantitative differences in proteins between AD eSEVs and AD eLEVs. Y-axis is expressed as the –Log (*P*-value) and x-axis as the difference (AD eLEVs-AD eSEVs). The DEPs were visualized above the black horizontal line at y =  − log (0.05) = 1.3. The dashed black lines represent the s0 of ± 0.1. The gene names of the 10 proteins with the highest fold change are indicated in blue and green for AD eSEVs and AD eLEVs, respectively. GWAS-associated proteins (CLU, APOE, CTSB and TSPAN14) are indicated in orange and some EV-associated proteins in red [1]. **c-f** GOBP analysis was performed on unique and DEP proteins and is represented as horizontal bar graphs for the 10 GOBPs with the highest -log(*P*-value). **c, d** GOBP of AD eSEVs unique proteins indicate implication in the integrin signaling, while AD eLEV unique proteins are related to the respiratory electron transport. **e, f** 139 from the 726 common proteins were found overexpressed in AD eSEVs with GOBP revealing a contribution to the translation processes. 336 proteins were found overexpressed in AD eLEVs of which GOBP analysis revealed numerous pathways related to platelet activation and brain immunity. These results suggest that there is an AD EV sub-population protein fingerprint related to specific functional implications. For **a** to **f**, BD-EVs were from a pool of 4 AD and eSEVs are represented in blue and eLEVs in green

We performed a GOBP analysis on our collagenase protein data. GOBP comparison between CTRL and AD patients demonstrated the presence of an AD signature in both AD-derived eSEVs and eLEVs (Fig. S6). We identified 50 proteins unique to AD eLEVs compared to CTRL eLEVs. None of these overlapped with the AD-unique proteins reported in [40] (Table S9), indicating that their AD-specific proteins were not unique in our dataset. Nevertheless, this showed that the EV content is clearly modified during the pathological course of AD. Knowing this, a proteomic comparison of AD eSEVs and AD eLEVs was done to assess whether both EV sub-populations have specific protein profiles allowing to further precise their own roles in AD pathophysiology (Fig. 4). We found 22 proteins unique to AD eSEVs (including 2 TM proteins agrin [AGRN] and sortilin [SORT1]) and 428 proteins (including 64 TM proteins) unique to AD eLEVs (Fig. 4a).

We then evaluated the differentially expressed proteins between CTRL and AD. We found 326 proteins overexpressed in AD eLEVs in comparison to CTRL, and 170 overexpressed in AD eSEVs compared to CTRL, from which two proteins (Annexin A [ANXA]1 and ANXA5) are also overexpressed in AD EVs ([40]; Table S9). After this, we compared AD eSEVs to AD eLEVs and show that within the 726 common proteins (Fig. 4a), 139 proteins were overexpressed in AD eSEVs and 336 proteins were overexpressed in AD eLEVs. The common proteins among AD eSEVs and AD eLEVs are visualized in a volcano plot where gene names are shown for the 10 proteins showing the highest fold change, EV-associated proteins and GWAS-associated proteins. GWAS-associated proteins along with known EV size-markers indicate that these proteins might help stratify vesicles. As such, CLU and APOE are significantly enriched in AD eSEVs along with CD9, CD81 and ITGB1. On the other hand, Cathepsin B (CTSB) along with ALIX, ANXA2, ITGA1, FLOT1 and FLOT2 are more present in AD eLEVs (Fig. 4b). Although common proteins exist between both populations, these data highlight the existence of specifically enriched proteins in both EVs populations.

We next compared AD eLEVs to AD eSEVs to identify the biological processes specifically enriched in each vesicle subtype. The GOBP analysis indicated that the unique AD eSEV proteins are mainly linked to the integrin signaling (Fig. 4c, Table S6) and the AD eSEV overexpressed proteins are more related to translation (Fig. 4e, Table S7). On the other hand, unique AD eLEVs proteins are more connected with the respiratory electron transport (Fig. 4d, Table S6), and their overexpressed proteins show an implication in the neuro-immune system (e.g. platelet activation, S1P pathways) (Fig. 4f, Table S7) [42]. Therefore, we defined the cellular origin of our EVs based on cell type-enriched proteins of EVs derived from hiPSC [43]. We observed an increase in glial origin of both eSEVs and eLEVs in AD conditions and a significant loss in neuronal eLEVs of AD (Fig. S7).

Therefore, we conclude that AD eSEVs and AD eLEVs have unique proteins and differentially expressed proteins involved in different biological pathways.

### Assessment of tau-seeding capacity of AD-derived EVs reveals a higher involvement of eSEVs in the seeding process

**Fig. 5:**
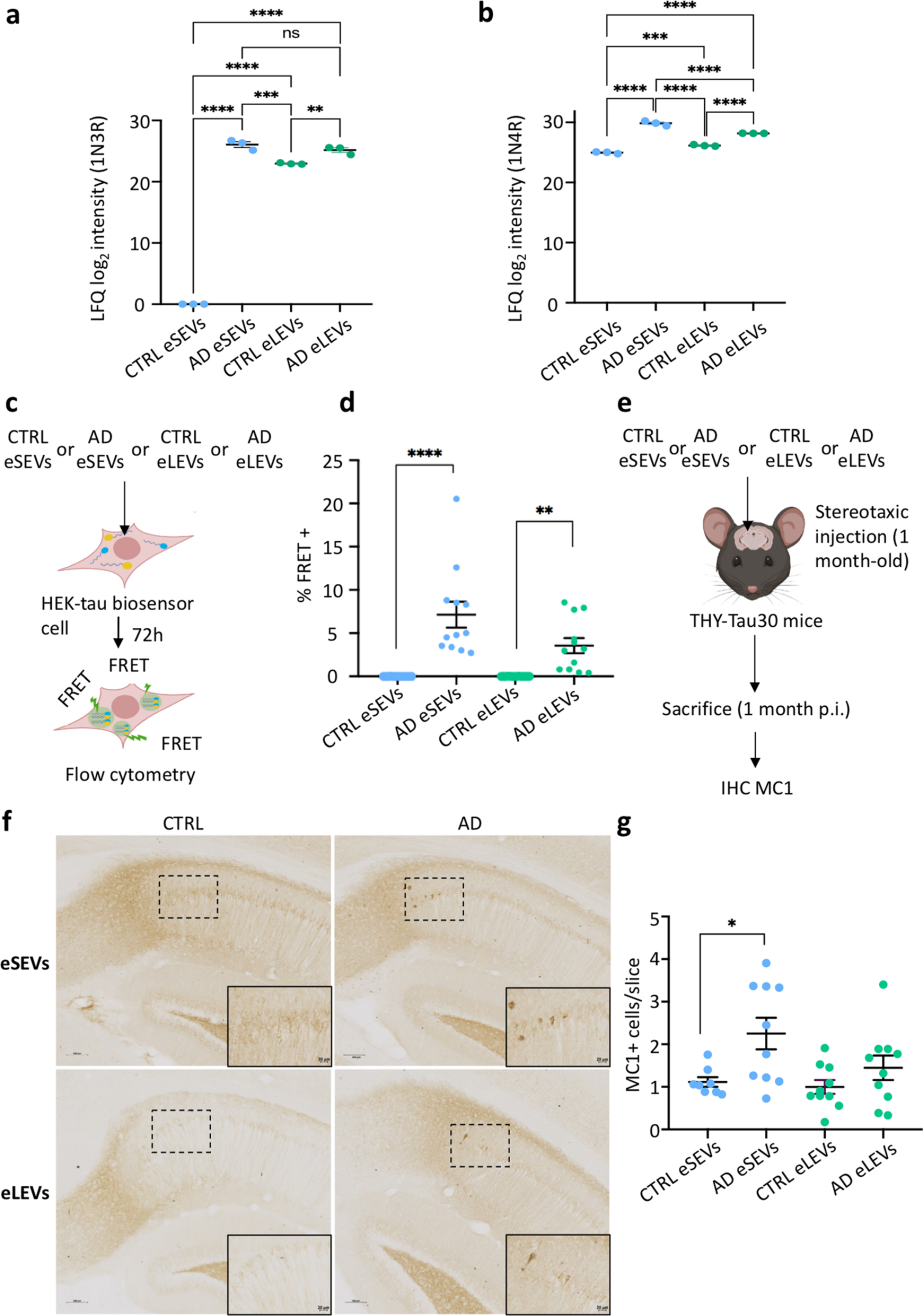
In vitro and in vivo studies of the tau seeding capacity of eSEVs and eLEVs. **a, b** 1N3R **(a)** and 1N4R **(b)** tau species detected by mass spectrometry in eSEVs and eLEVs of CTRL and AD patients. Dots correspond to 3 technical replicates of BD-EVs from a pool of CTRL (3 dots) or 3 technical replicates of BD-EVs from a pool of AD (3 dots).** c** Schematic representation of the HEK-tau FRET biosensor cell model used to study in vitro seeding capacity of EVs. **d** Dotplot indicating the percentage of FRET-positive cells reveals the presence of tau seeds inside AD eSEVs and AD eLEVs in comparison to their respective controls. Three independent experiments were done for eSEVs and eLEVs of four individual AD patients or four individual CTRLs. Kruskal–Wallis multiple comparison, **P < 0.01, ****P < 0.0001. **e** Schematic representation of the bilateral stereotaxic injection of CTRL or AD eSEVs or eLEVs into the hippocampus of one-month-old THY-Tau30 transgenic mice. p.i.: post-injection. **f** Representative images of the immunostaining of MC1 in the hippocampus (injection site). Scale bar, 100 µm; zoomed images, 20 µm. **g** Dotplot representing the number of MC1-positive cells per brain slice for CTRL (pool of 4 CTRL) and AD (pool of 4 AD) eSEVs and eLEVs. *n* = 4 mice for control eSEVs corresponding to 8 dots (one per hemisphere) and *n* = 5 mice for control eLEVs, AD eSEVs and AD eLEVs corresponding to 10 dots (one per hemisphere). Blinded quantification indicates a significant increase of tau seeding capacity of AD eSEVs. Ordinary one-way ANOVA, parametric comparison of all four groups. *P < 0.05

The distinct protein signatures found in AD eSEVs and AD eLEVs could indicate specialized functional roles. Therefore, we selected tau seeding as a readout of these specialized functions and compared the ability of AD eSEVs and AD eLEVs to induce tau nucleation in vitro and in vivo. First, quantification of tau species by proteomics revealed that loading of 1N3R tau in AD eSEVs was induced by the pathology as it was not found in CTRL eSEVs (Fig. 5a). Also, AD eSEVs were significantly enriched with 1N4R tau compared to all other groups (Fig. 5b).

AD eSEVs, AD eLEVs, CTRL eSEVs and CTRL eLEVs were lipofected into the HEK-tau FRET biosensor cells (Fig. 5c). The flow cytometry analysis enabled quantification of the percentage of FRET-positive cells after 72-h incubation. The results indicate that both subtypes of AD EVs contain seeds in comparison to their respective CTRLs (Fig. 5d). This HEK-tau FRET cellular model requires the use of lipofectamine for EV internalization within the HEK biosensor cells. This circumvents differences in cellular internalization affinity to EV sub-populations. The EV-mediated prion-like propagation hypothesis implicates both a successful cellular internalization of the EVs and a high seed-competent content. Hence, we used the THY-Tau30 transgenic mouse model to assess the in vivo seeding capacity including both EV cellular uptake affinity and seeding capacity [38]. For this, one-month-old THY-Tau30 mice received hippocampal bilateral stereotaxic injections of 1.6 × 10^9^ eSEVs or eLEVs from CTRL or AD patients (Fig. 5e). At the time of injection, these mice have low endogenous tau pathology, allowing the evaluation of the ability of EVs to induce tau nucleation in vivo. The MC1 immunostaining was used to visualize misfolded tau lesions within neurons in CA1 (Fig. 5f) [39]. Blinded MC1^+^ cell quantification indicated that injection of CTRL eSEVs and eLEVs led to a low level of basal MC1^+^ staining inherent to the THY-Tau30 model. On the contrary, injection of AD-derived eSEVs showed a higher in vivo seeding capacity, which was not observed for AD eLEVs (Fig. 5g).

Taken together, we demonstrated here that whereas both AD eSEVs and AD eLEVs contain seeds, only AD eSEVs are prone to mediate tau nucleation in a prion-like manner.

## Discussion

In this study, we explored the biological pathways and functional implications of AD EV sub-populations to assess their roles in AD pathophysiology and define new and specific EV markers in AD condition. For this, we effectively stratified eSEVs and eLEVs from human BDF using a size-based separation protocol combining SEC and UC. The SEC efficiently removes soluble proteins of the interstitial fluid, including free tau and other pathological proteins, from EVs to enable specific study of BD-EVs in AD. The subsequent differential UC separates eLEVs and eSEVs based on sedimentation coefficient, which is size-dependant [4].

Here, collagenase type 3 was identified as the optimal dissociation enzyme for preserving EV integrity and enriching EV-specific proteins while minimizing multivesicular body contamination. Although papain has been widely used [44–46], recent findings, including ours and other retrospective research [47], highlight superiority of collagenase in enhancing EVs purity. We compared collagenase and papain dissociation on the same frozen brain samples to circumvent differences in sample origin, EV isolation method or inter-laboratory bias. Both enzymes showed low cellular contamination based on the MISEV2023 [4], with collagenase showing an enrichment in EV-associated proteins (TM and non-TM proteins) compared to papain. Whereas classical markers of EVs are found in our small and large EVs, some of them were missing (CD63, TSG101). The absence of these markers may be linked to technical issues or to different addressing of these proteins in EVs under AD conditions. Also, knowing that currently there is a lack of universal molecular markers of ectosomes, exosomes or other EV subtypes [4], we identified proteins that better differentiate eSEVs from eLEVs than classical EV markers. This indicates the importance of defining markers of specific EVs (and EV subtypes) for a pathological or physiological condition, which are crucial not only for investigating pathological mechanisms but also for discovering new molecules for diagnosis.

Unlike papain, collagenase specifically targets peptides with repeating sequence glycine-proline-hydroxyproline mainly present in proteins of the extracellular matrix such as collagen and elastin, enabling TM proteins to be better preserved. These TM proteins, enriched by collagenase, are essential for EV classification, immunoprecipitation and functional studies, particularly in the context of AD where EV surfaceomes may influence cell vulnerability. We demonstrated that the dissociation enzyme significantly affects EV quality and recommend collagenase type 3 for standardized characterization of human BDF EVs. This finding emphasizes the importance of harmonizing protocols to improve reproducibility across studies of BD-EVs.

We then investigated implications of AD EV sub-populations in AD pathology and consider our work as a first step to highlight common and conserved phenomena among AD that might imply EVs. Pooling biological samples prior to MS allows identification of common mechanisms, opening up new avenues that should be investigated in individual patients, in Braak-stratified patients, in rapid or low decliners, etc. Also, a very important area of research regarding EVs is the identification of clusters. An initial pool can be used to define the different EV sub-populations present in biofluids, after which the identification of these EV subtypes across individual patient samples can be done to evaluate inter-patient variability.

EVs transport proteins central to AD pathogenesis, including Aβ, tau and APOE [21, 23, 40, 51]. We therefore evaluated the presence of GWAS-associated proteins in BD eSEVs and eLEVs. Notably, APOE—the most well-known AD GWAS-associated protein—was detected under all conditions and showed a trend toward increased levels in AD eLEVs compared to control eLEVs (*P* = 0.063). Clusterin (CLU/APOJ), an extracellular chaperone (particularly for tau [52]) and established AD risk gene [41], was significantly elevated in both eLEVs and eSEVs from AD patients, as well as in the brain parenchyma (*P* = 0.057). This suggests that the sorting of CLU into BD-EVs from AD patients may result from its over-accumulation in neural cells. Previous studies have reported increased CLU levels in the hippocampus, frontal cortex [53], and CSF of AD patients [54]. Additionally, FERMT2 was elevated in eLEVs (*P* = 0.0545). While FERMT2 mRNA overexpression has been observed in the post-mortem temporal cortex of AD patients [55], other studies did not detect such changes in post-mortem brain tissue [56], consistent with our findings.

CTSH was uniquely detected in AD eLEVs, and TSPAN14 was found only in AD EV subtypes (Fig. 3a). The relationship between these proteins and tau propagation, as well as their transport within EV sub-populations, remain to be elucidated. Recent evidence suggests that CTSH (and CTSB) and TSPAN14 may modulate microglial activation [57–59], aligning with dysregulated pathways identified in our proteomic analysis, such as astrogliosis, brain immunity, and microglial activation. While some studies have identified Aβ and APP in a whole pool of AD BDF-derived EVs [40], APP was surprisingly not detected in our proteomic study. This could be due to technical limitations related to differences in sample preparation protocol or biological sample variability (including donor age, sex or brain region). In any case, a cross-study comparison highlights a significant amount of overlap (Table S9).

To further characterize the proteomic signatures of AD eSEVs and eLEVs, we performed unbiased proteomic profiling. Previous studies have examined protein profiles of whole BD-EVs population, but mainly in control subject [27, 40, 46, 48–50]; by contrast, here we investigated proteomic profiles of specific EV sub-populations, revealing that AD eSEVs are associated with biological pathways related to integrin signaling at the synapse. Integrin signaling involves ligand-binding and downstream molecular activations that contribute to both brain immunity and formation of the focal adhesion (FA) complex at synapses [57]. The FA complex, triggered by Aβ, is linked to astrogliosis, microglial activation, and increased tau phosphorylation in AD [57]. In contrast, AD eLEVs were enriched for pathways involved in respiratory electron transport and brain immunity (platelet activation and S1P pathways). The electron transport chain, a central component of mitochondrial metabolism, has previously been reported to be enriched in BD-EVs from AD patients [40]. Our subpopulation analysis refines these findings, implicating AD eLEVs in mitochondrial dysfunction—a hallmark of AD [60].

The detection of brain-immunity-related GOBPs may reflect the increased glial origin of both eSEVs and eLEVs in AD, alongside a loss of neuronal eLEVs (Fig. S7). This observation is consistent with the advanced Braak stage VI of the AD brain samples used in our study. Future research should map brain area-dependent EV content, as initiated by Huang et al. for healthy individuals, and correlate these findings with different pathological stages of AD [50].

Our biological pathway assessment revealed distinct pathways within the protein content of AD eSEVs and eLEVs, suggesting unique roles for each subtype in AD pathophysiology. To investigate this further, we compared the seeding capacity of AD-derived eSEVs and eLEVs. While both subtypes contained tau seeds, AD eSEVs exhibited a higher in vivo seeding/propagation capacity (Fig. 5). Importantly, eSEVs were approximately 10 times more abundant than eLEVs in human BDF, as quantified by NTA (Fig. 1b). Thus, the injection of equal numbers of AD eSEVs and eLEVs in vivo demonstrated a greater seeding capacity for eSEVs, supporting their predominant role in tau pathology propagation.

We also observed that 1N4R tau species were present in both CTRL- and AD-derived eLEVs and eSEVs, whereas 1N3R tau species were specific to AD eSEVs. These proteoforms may underlie the enhanced seeding capacity of AD eSEVs. Future studies should employ tau-targeted proteomics to map the tau proteoforms within AD EV subtypes.

AD eSEVs emerge as a promising therapeutic target due to their high seeding capacity and abundance. In contrast, AD eLEVs—which also contain tau seeds—may play a dual role: either facilitating the clearance of immunity-triggering proteins or stimulating brain immunity. AD eLEVs are enriched in immune-related proteins (Fig. S6b), potentially enhancing their internalization by glial cells and promoting clearance. Alternatively, these proteins could act as chemokines, further activating brain immunity. Notably, AD eLEVs contain proteins linked to platelet activation and FERMT2, a GWAS protein and mediator of the FA complex [61], which is known to stimulate astrogliosis and microglial activation.

EV-mediated seeding is influenced by multiple factors beyond tau proteoforms. TM proteins, such as AGRN and SORT1—exclusively detected in eSEVs—may contribute to the higher seeding capacity of AD eSEVs. AGRN is a syndecan TM protein, and syndecans have been shown to facilitate EV internalization [62, 63] and are implicated in AD pathophysiology [64–67]. Additionally, co-factors transported within EVs, such as RNA [68], lipids (e.g., cholesterol) [69], or proteins (e.g., CLU) [52], may shield and enhance tau seeding. We cross-referenced our data with the tau interactome [70–75] and tau seeding modifiers [76], identifying 17 proteins (including CLU and BIN1) common to these databases (Table S3). These proteins represent potential co-factors driving tau propagation within EVs.

Our findings highlight distinct roles for AD eSEVs and eLEVs in tau propagation and immune modulation. Further investigation into the relationship between the proteomic signatures and functional roles of EV subtypes will deepen our understanding of their pathophysiological implications in AD.

## Conclusion

Overall, our results show that collagenase-derived brain EV sub-populations from AD patients, separated based on size, indicate specific protein profiles. Importantly, we found that AD eSEVs have a higher tau seeding capacity in vivo, highlighting their significant contributions to tau propagation and providing new insights into the different roles of EV sub-populations in AD.

## Supplementary Information

Additional file 1. **Fig. S1**. New size-based EVs separation protocol of BDF using papain dissociation. **Fig. S2**. NTA measurement of EV concentration after combining SEC to UC for the eSEV and eLEV isolation. **Fig. S3**. eLEVs and eSEVs from AD and CTRL patients. **Fig. S4**. EV markers. **Fig. S5**. Detection of CLU and FERMT2 in EVs and brain lysate (BL). **Fig. S6**. GOBP comparison of collagenase-derived eSEVs and eLEVs between non-demented controls (CTRL) and AD. **Fig. S7**. Cellular origin of eSEVs and eLEVs from control and AD patients.

Additional file 2. **Table S1**. Overview table of the used human samples for each experiment. **Table S2**. Proteomic analysis of eSEVs and eLEVs from AD and controls prepared using papain for brain dissociation. **Table S3**. Proteomic analysis of eSEVs and eLEVs from AD and control prepared using collagenase type 3 for brain dissociation. **Table S4**. List of proteins from MISEV2023 guidelines. **Table S5**. GWAS-associated proteins differentially expressed among non-demented controls (CTRL) and AD patients. **Table S6**. List of gene ontology biological pathways (GOBP) of unique proteins. **Table S7**. List of gene ontology biological pathways of AD overexpressed proteins. **Table S8**. EVs-associated proteins detected in papain or collagenase derived EVs. **Table S9**. Comparison of proteomics data (collagenase) with BD-EV proteomics from Muraoka and collaborators (papain).

Additional file 3. Full uncropped blots for Fig. 3 and Fig. S5.

## Data Availability

The datasets used and/or analyzed during the current study are available from the corresponding author on reasonable request. The mass spectrometry proteomics data have been deposited to the ProteomeXchange Consortium via the PRIDE [33] partner repository with the dataset identifier PXD055734.

## Abbreviations

AGRN: Agrin
AD: Alzheimer’s disease
BD-EVs: Brain-derived EVs
BDF: Brain-derived fluid
CSF: Cerebrospinal fluid
DEP: Differentially Expressed Proteins
CLU: Clusterin
EVs: Extracellular vesicles
eSEVs: Enriched-small EVs
eLEVs: Enriched-large EVs
FA: Formic acid
FRET: Fluorescence resonance energy transfer
GOBP: Gene Ontology biological processes
GOCC: Gene Ontology cellular components
GWAS: Genome-wide association study
hiPSC: Human induced Pluripotent Stem Cell
IHC: Immunohistochemistry
LFQ: Label-free quantification
LC–MS/MS: Liquid Chromatography coupled to tandem mass spectrometry
MISEV: Minimal Information for Study of Extracellular Vesicles guidelines
non-TM: Luminal non-transmembrane
NTA: Nanoparticle tracking analysis
CTRL: Non-demented controls
PCA: Principal component analysis
SEC: Size-exclusion chromatography
SNPs: Single nucleotide polymorphisms
TM: Transmembrane
TEM: Transmission electronic microscopy
UC: Ultracentrifugation
