# Supplementary material for: Stratification of brain-derived extracellular vesicles of Alzheimer’s disease patients indicates a unique proteomic content and a higher seeding capacity of small extracellular vesicles
Source: Transl Neurodegener. 2025 Dec 5;14:63. doi: 10.1186/s40035-025-00519-z (PMC12679798; doi:10.1186/s40035-025-00519-z)
Supplement: Supplementary file 1 — Additional file 1. Fig. S1. New size-based EVs separation protocol of BDF using papain dissociation. Fig. S2. NTA measurement of EV concentration after combining SEC to UC for the eSEV and eLEV isolation. Fig. S3. eLEVs and eSEVs from AD and CTRL patients. Fig. S4. EV markers. Fig. S5. Detection of CLU and FERMT2 in EVs and brain lysate (BL). Fig. S6. GOBP comparison of collagenase-derived eSEVs and eLEVs between non-demented controls (CTRL) and AD. Fig. S7. Cellular origin of eSEVs and eLEVs from control and AD patients. [file 40035_2025_519_MOESM1_ESM.docx]

**Supplemental Information**

Supplementary Figures S1-7

**Stratification of Brain-Derived Extracellular Vesicles of Alzheimer’s Disease Patients Indicates a Unique Proteomic Content and a Higher Seeding Capacity of Small Extracellular Vesicles**

Marie Oosterlynck^1,†^, Elodie Leroux^1,†^, Balasubramaniam Namasivayam^1^, Thomas Bouillet^1^, Raphaelle Caillierez^1^, Anne Loyens^1^, Daniele Mazur^1^, Romain Perbet^1^, Christophe Lefebvre^2^, Soulaimane Aboulouard^2^, Claude-Alain Maurage^3^, Bertrand Accart^3^, Luc Buée^1*^ and Morvane Colin^1*^

1. Univ. Lille, Inserm, CHU-Lille, Lille Neuroscience & Cognition, F-59000 Lille, France.

2. Univ. Lille, Inserm, U1192-Laboratoire Protéomique, Réponse Inflammatoire et Spectrométrie de Masse (PRISM), Lille, France.

3. Univ. Lille, CHU-Lille, CRB/CIC1403, Centre de Ressources Biologiques du Centre d’Investigation Clinique, Lille, France.

†. Equal contributors

* Corresponding authors:

Drs M. Colin & L. Buée

Lille Neuroscience & Cognition, Inserm UMR-S 1172, ‘Alzheimer & tauopathies’

Univ. Lille, Fac. of Medecine – pole recherche

Bâtiment Biserte, rue Polonovski

59045 Lille Cedex, France

Tel: 33-3-20 62 20 73, Fax: 33-2-20 53 85 62

M.C. (morvane.colin@inserm.fr, ORCID 0000-0003-0611-4167) and L.B. (luc.buee@inserm.fr, *ORCID* 0000-0002-6261-4230)

**
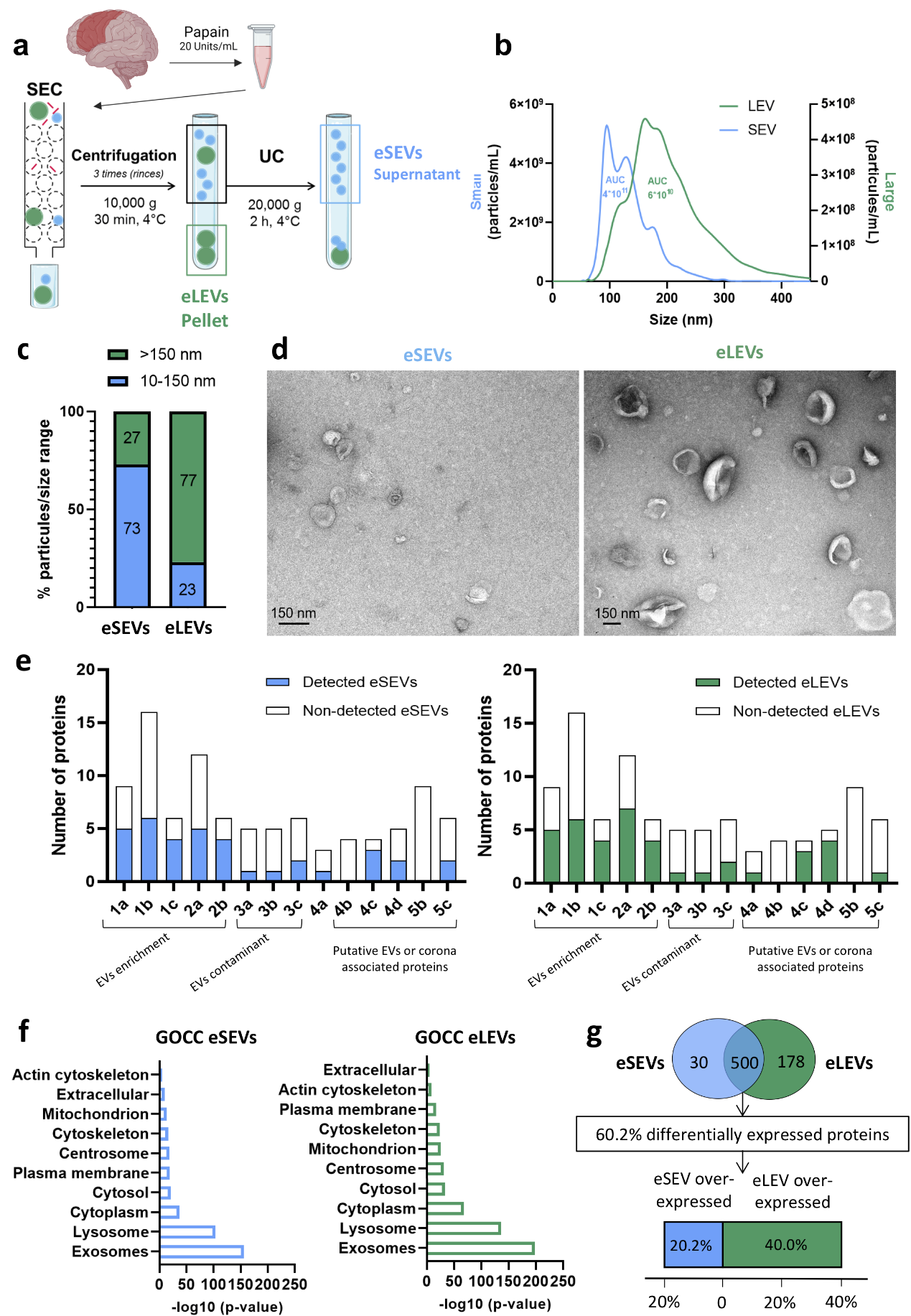
****Fig. S1.** **New size-based EVs separation protocol of BDF using papain dissociation. a** BDF is obtained by papain enzymatic dissociation as previously done for AD and CTRL brain extracts [21]. BDF were loaded on top of a sepharose SEC column and EVs were separated in PBS. Consecutive centrifugation steps enable separation of enriched large EVs (eLEVs: >150 nm) an enriched small EVs (eSEVs: 10-150 nm). **b** Size distribution of eSEVs and eLEVs was determined by NTA analysis. Data are presented as the mean of 2 individual CTRL and one AD. **c** eSEVs and eLEVs separation enrichment was calculated from NTA and indicate a 73% and 77% enrichment in eSEVs and eLEVs fraction, respectively. **d** eSEVs and eLEVs morphology was visualized by TEM. Scale bar = 150 nm. Images are coming from a pool of 2 CTRL- and 1 AD-derived BD-EVs. **e** Vertical bar graph corresponding to the number of proteins detected in eSEVs and eLEVs as recommended by the MISEV2023 guidelines after mass spectrometry-based proteomic analysis [3]. MISEV2023 categories; 1a: Multi-pass TM proteins associated with plasma membrane and/or endosomes; 1b: Single-pass TM proteins associated with plasma membrane and/or endosomes; 1c: GPI-or lipid-anchored proteins associated with plasma membrane and/or endosomes; 2a: Cytosolic proteins with lipid or membrane protein-binding ability; 2b: Cytosolic proteins with promiscuous incorporation into EVs; 3a: Lipoproteins; 3b: Protein and protein/nucleic acid aggregates; 3c: Exomere or supermere-enriched components; 4a: Nucleus; 4b: Mitochondria; 4c: Secretory pathway: Endoplasmic reticulum, Golgi apparatus; 4d: Autophagosomes, cytoskeleton; 5b: Cytokines and growth factors; 5c: Adhesion and extracellular matrix proteins. **f** Horizontal barplot of –log (*P*-value) from LFQ intensities obtained for 10 selected GOCC terms after quantitative proteomic analysis of eSEVs (left) and eLEVs (right). **g** Venn diagram (top) indicating unique proteins for both eSEVs and eLEVs as well as 500 common proteins of which 60.2% were found differentially expressed. A boxplot represents the repartition of differentially expressed proteins between eLEVs and eSEVs (bottom). For (**e**) to (**g**) EVs are coming from a pool of 4 CTRL and from a pool of 4 AD (post-mass spectrometry fusion). For (**a**) to (**g**), eSEVs are represented in blue and eLEVs in green.

**
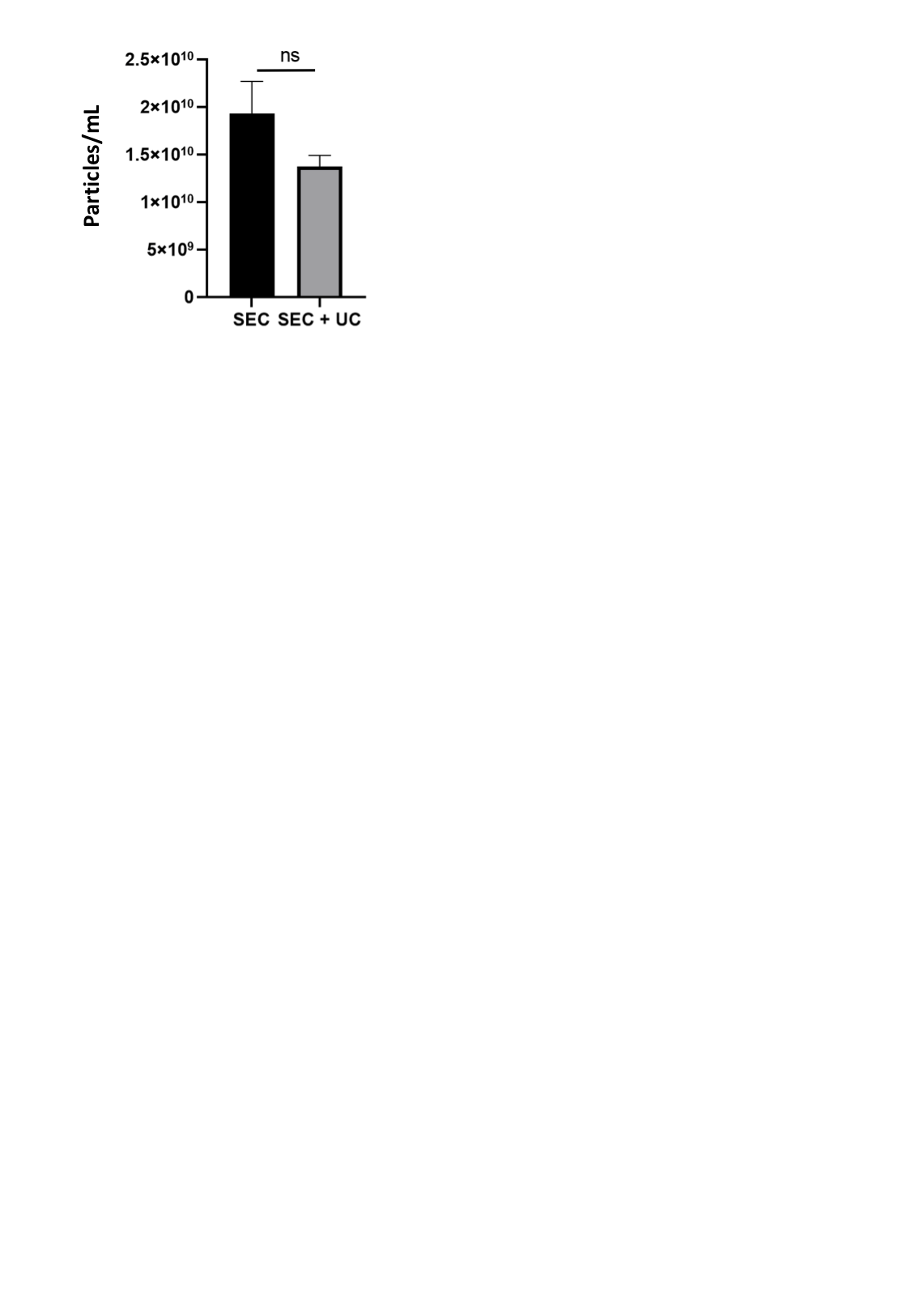
****Fig. S2. NTA measurement of EVs concentration after combining SEC to UC for the eSEVs and eLEVs isolation.** Human BD-EVs from papain enzymatic brain dissociation were quantified after SEC followed or not by a 20,000x g UC. Measures were done by NTA and expressed in particles/mL. No significant EVs loss was observed in the SEC combined to UC protocol. Data are presented as BD-EVs concentration (particles/mL) of 2 individual CTRL and 1 individual AD, analysed in triplicate. Mann-Whitney test, non-parametric.


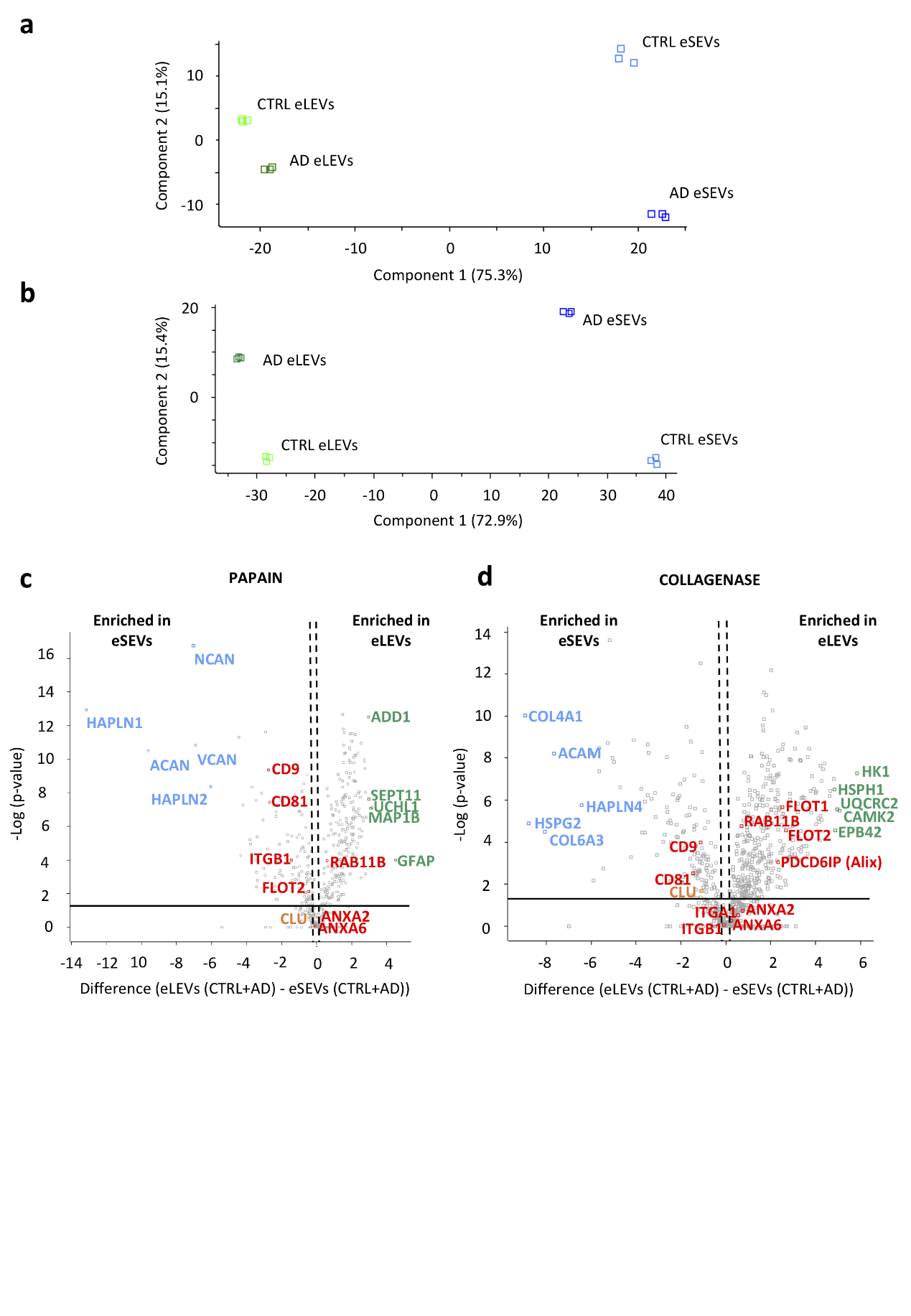


**Fig. S3. eLEVs and eSEVs from AD and CTRL patients. a-b** eSEVs and eLEVs derived from a pool of 4 AD or a pool of 4 CTRL were isolated after papain or collagenase brain dissociation. Three technical replicates of these pools were analyzed by mass spectrometry. The proteomic data were further treated in Perseus. PCA was done and revealed a successful separation for both papain (**a**) and collagenase (**b**). **c-d** Volcano plot of quantitative differences in proteins between eSEVs (CTRL+AD) and eLEVs (CTRL+AD) prepared from papain (**c**) or collagenase (**d**) digestion after a Student’s t-test with a p-value set to 0.01 (Perseus software). Y-axis is expressed as the –Log (p-value) and x-axis as the difference (eLEVs – eSEVs). The DEP were visualized above the black horizontal line at y = −log (0.05) = 1.3. The dashed black lines represent the s0 of ±0.1. The gene name color code comprises of red for EVs-associated proteins [1]; blue for 5 proteins overexpressed in eSEVs (highest fold change); green for 5 proteins overexpressed in eSEVs (highest fold change) and orange for GWAS-associated proteins. CD9 and CD81 are overexpressed in eSEVs. For (**c**) to (**d**), BD-EVs are coming from a pool of 4 CTRL and a pool of 4 AD.


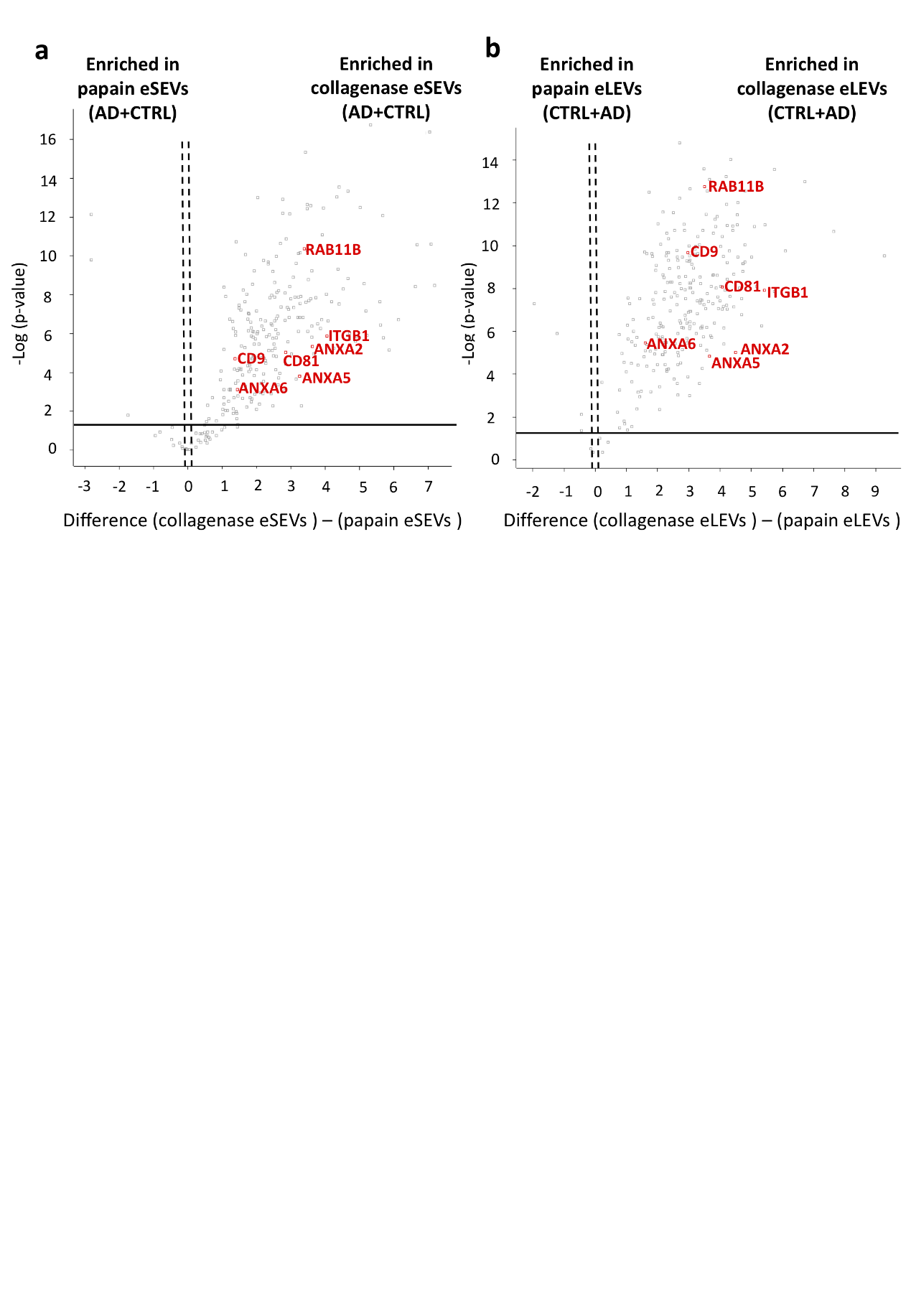


**Fig. S4. EV markers.** eSEVs and eLEVs derived from the papain or collagenase brain dissociation of CTRL and AD brain tissue were analysed by mass spectrometry. Volcano plots of quantitative differences in proteins between eSEVs (CTRL+AD) prepared from papain or collagenase digestion **(a)** and comparison of eLEVs (CTRL+AD) prepared from papain or collagenase digestion **(b)**. Y-axis is expressed as the –Log (*P*-value) and x-axis as the difference (collagenase-papain). The DEP were visualized above the black horizontal line at y = −log (0.05) = 1.3. The dashed black lines represent the s0 of ±0.1. The red gene name indicates some known EV-associated proteins [1]. BD-EVs are coming from a pool of 4 CTRL and a pool of 4 AD.


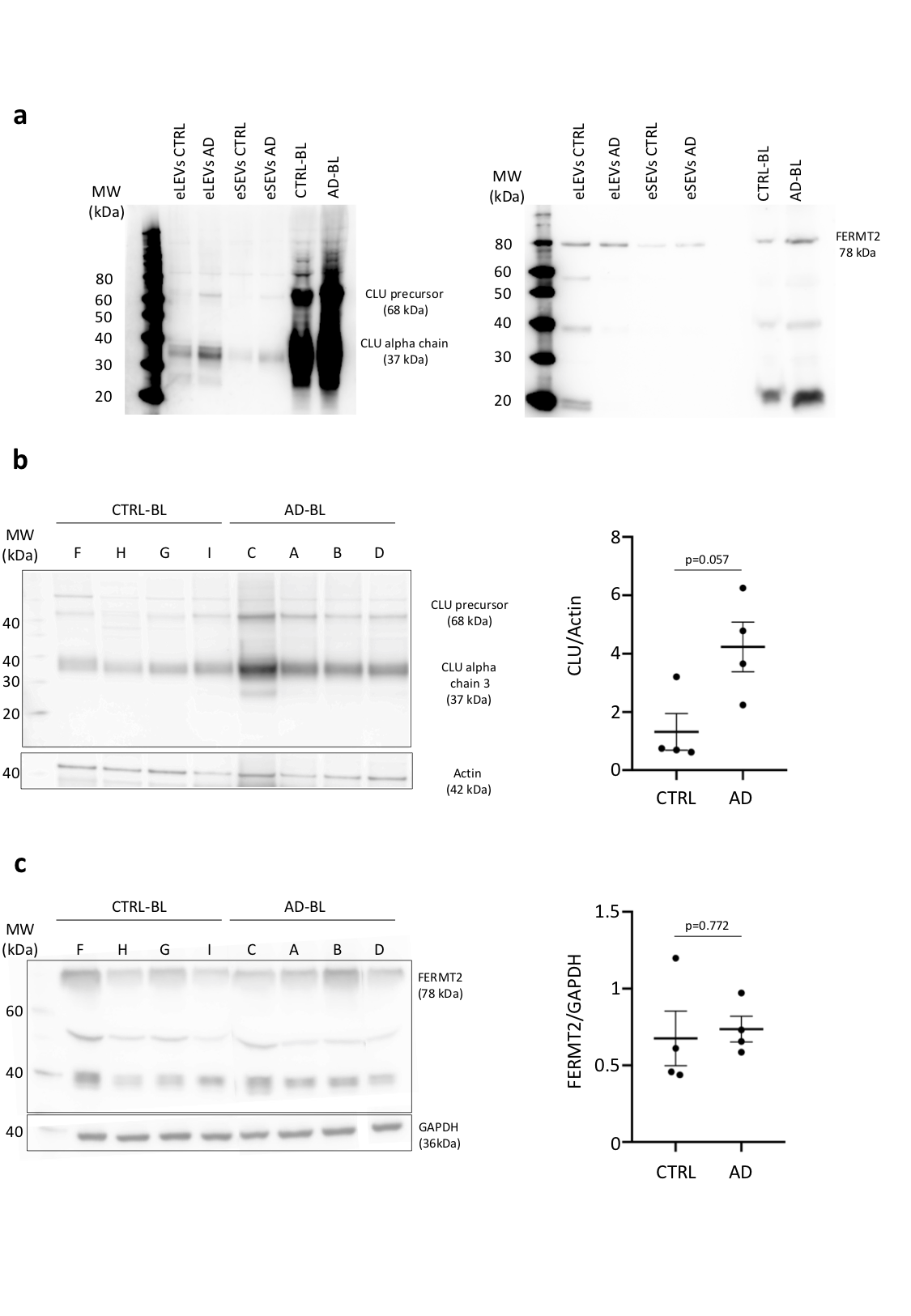
 **Fig. S5. Detection of CLU and FERMT2 in EVs and brain lysate (BL). a-b** 5x10^9^ eSEVs and eLEVs derived from collagenase brain dissociation of non-demented controls (CTRL, pool of 4 subjects) and AD (pool of 4 patients) brain tissue were loaded onto NuPage western blots. Whole blots are shown for (**a, left**) detection of clusterin (CLU) and (**a, right**) detection of FERMT2. **b-c** 10 µg of brain lysates of 4 individual CTRL and 4 individual AD were loaded onto NuPage western blots for (**b**) detection of clusterin (CLU) and (**c**) detection of FERMT2. The ratio CLU/Actin and FERMT2/GAPDH are shown in the right part of (**b**) and (**c**).


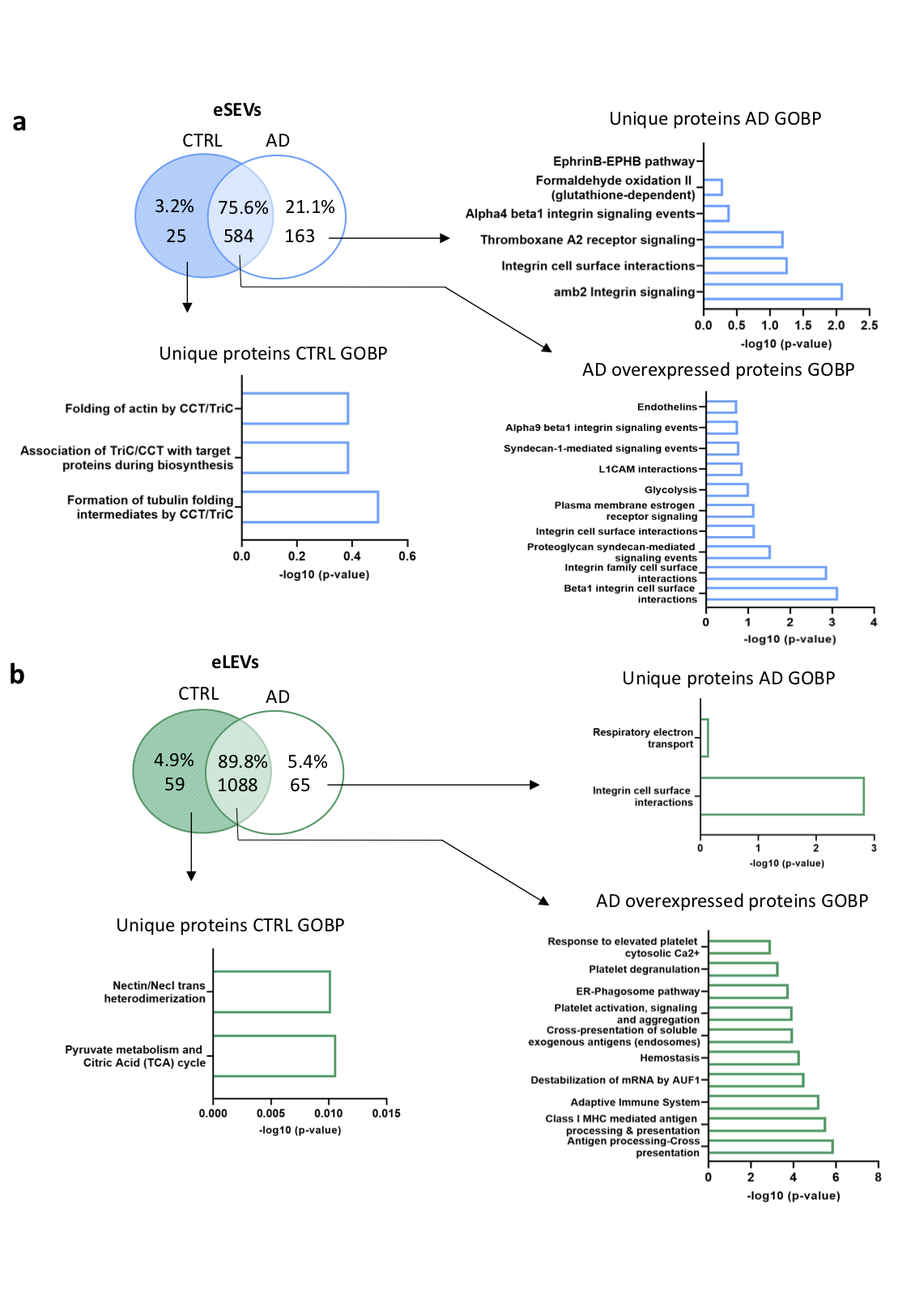
 **Fig. S6. GOBP comparison of collagenase-derived eSEVs and eLEVs between non-demented controls (CTRL) and AD.** Venn diagram indicating the number of unique and shared proteins between CTRL and AD eSEVs (**a**) and eLEVs (**b**). Gene ontology biological pathways (GOBP) of the unique proteins for CTRL eSEVs (**a**, left) and AD eSEVs (**a**, top right) are represented in horizontal bar graphs of the –log10(*P*-value). 163 proteins were found uniquely in AD eSEVs mainly implicated in integrin signalling (**a**, bottom right). GOBP of the unique proteins for CTRL eLEVs (**b**, left) and AD eLEVs (**b**, top right). 65 proteins were found uniquely in AD eLEVs and relate to the integrin and respiratory electron transport. GOBP of the 327 AD eLEVs overexpressed proteins relate to the brain-immunity (**b**, bottom right). For (**a**) and (**b**), BD-EVs are coming from a pool of 4 CTRL or a pool of 4 AD.


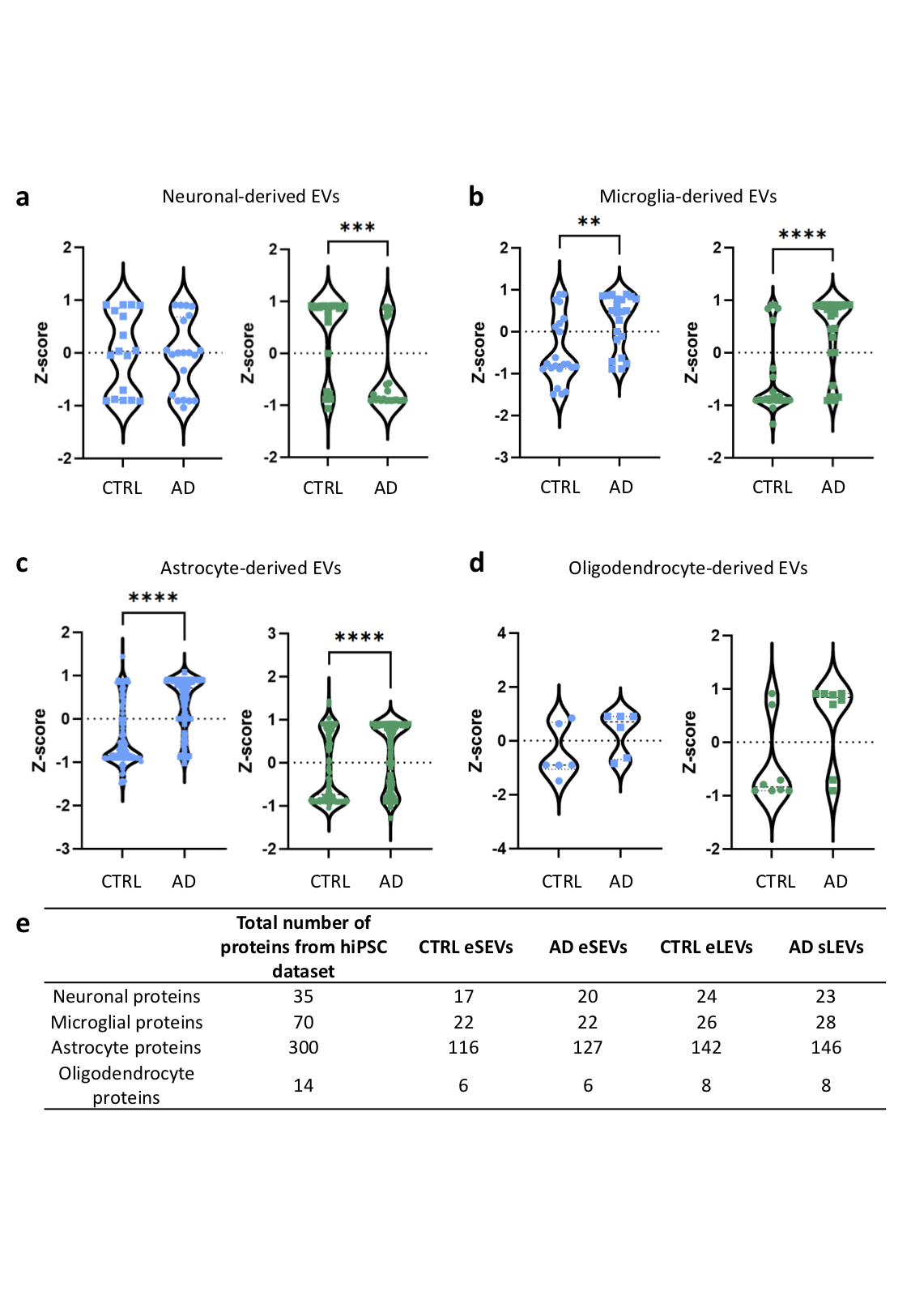
 **Fig. S7.** **Cellular origin of eSEVs and eLEVs from control and AD patients.** Proteomic data of eSEVs and eLEVs of a pool of 4 CTRL or a pool of 4 AD was crossed with the enriched proteins list of You and collaborators (2022) originating from EVs of hIPSC differentiated into different cell types [43]. Each dot represents a protein found in our database and the database of You and collaborators (2022). Violin plots of the proteins found for neuronal- (**a**), microglia- (**b**), astrocyte- (**c**) and oligodendrocyte (**d**)-derived eSEVs and eLEVs are shown. Mann-Whitney, non-parametric test comparing AD to CTRL. *P*-values are calculated on Z-score values. ***P* <0.01, ****P* < 0.001, *****P* < 0.0001. For (**a**) to (**d**), eSEVs are represented in blue and eLEVs in green. **e** List representing the number of eSEVs and eLEVs proteins found enriched for a particular cellular origin. The total number of proteins with a 5-fold enrichment of neuronal, microglial, astrocyte or oligodendrocyte origin from you et al. (2022) are shown in the first column [43]. For neuronal- and oligocendrocyte-EVs more than 50% of cell type enriched proteins were found, while this was lower for microglial and astrocyte enriched proteins.
