# Supplementary material for: Stratification of brain-derived extracellular vesicles of Alzheimer’s disease patients indicates a unique proteomic content and a higher seeding capacity of small extracellular vesicles
Source: Transl Neurodegener. 2025 Dec 5;14:63. doi: 10.1186/s40035-025-00519-z (PMC12679798; doi:10.1186/s40035-025-00519-z)

Full uncropped blots figure 3 with CTRL and AD brain lysate (BL)

a

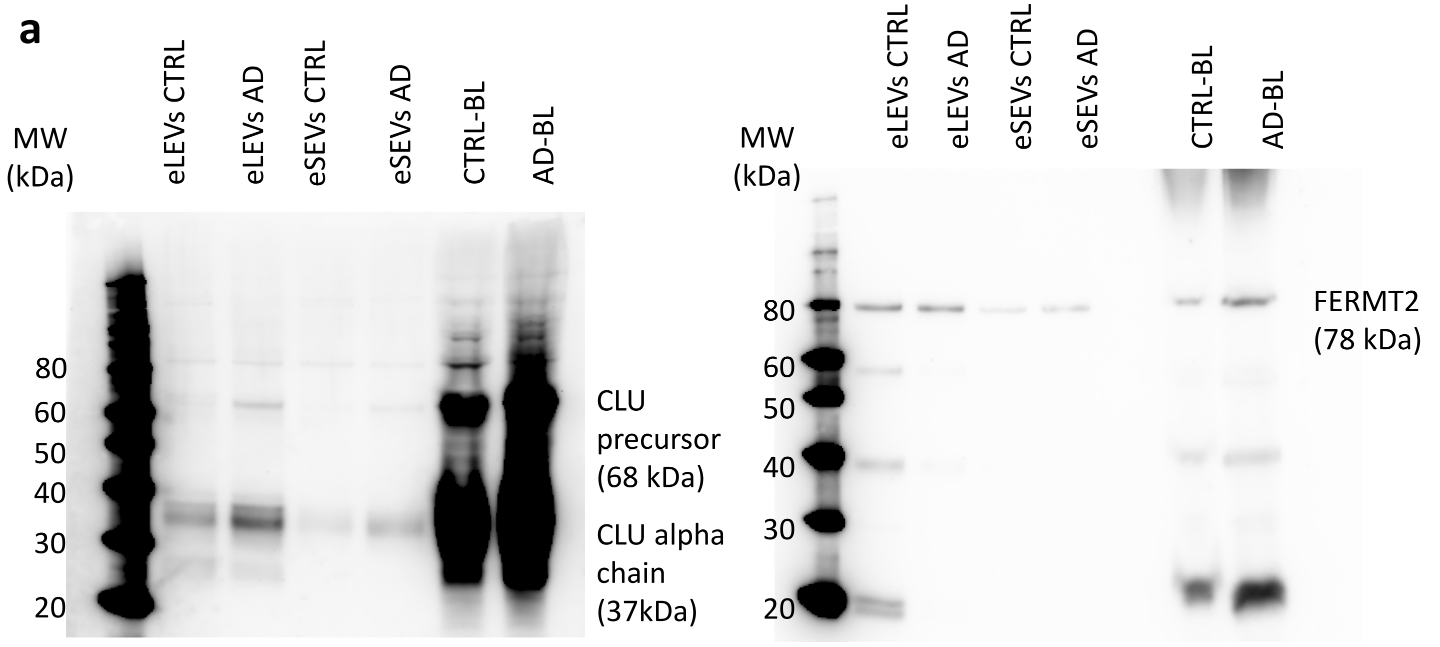

Full uncropped blots supplementary figure 5

b

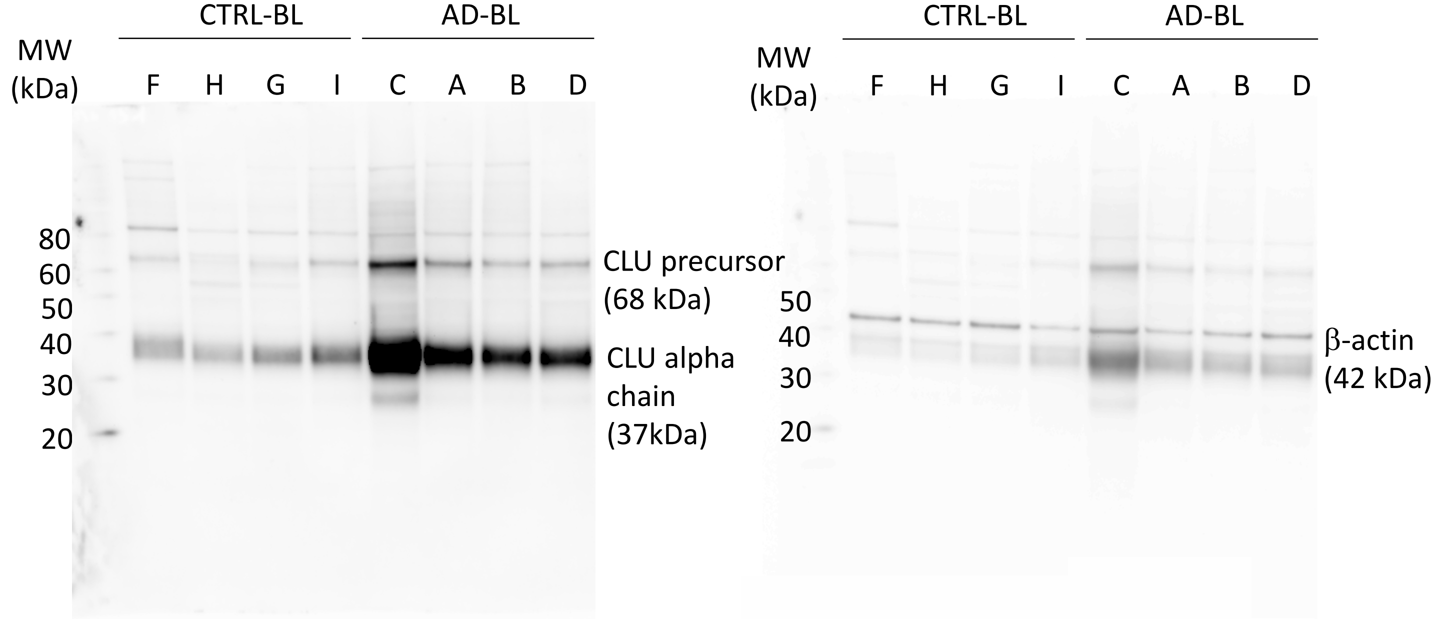

c

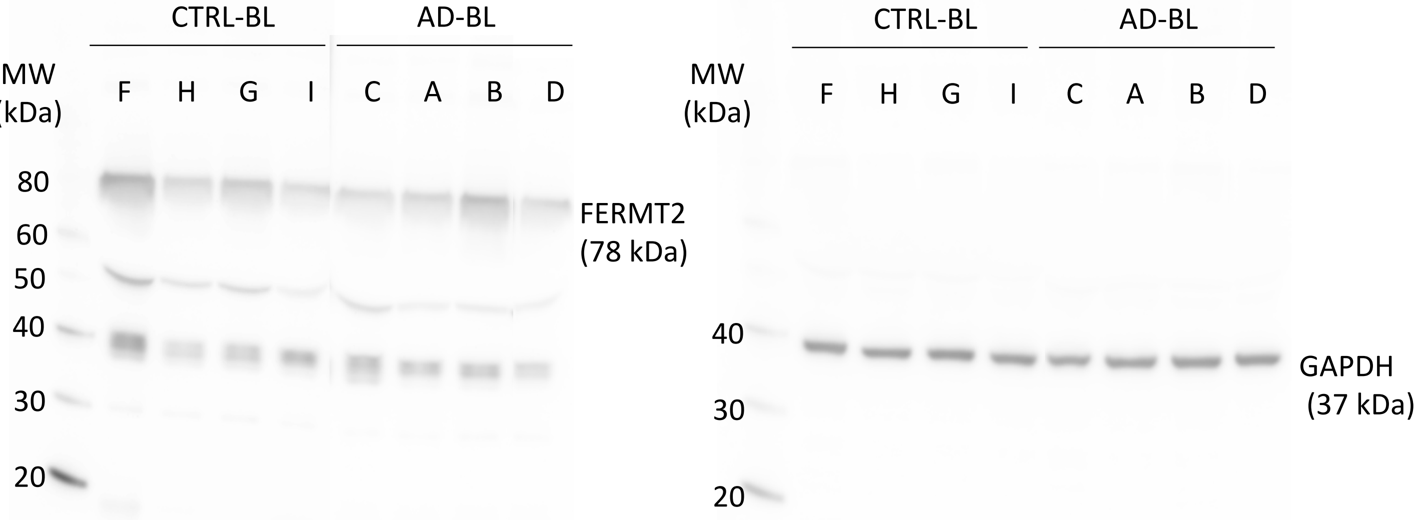

Supplement: Supplementary file 3 — Additional file 3. Full uncropped blots for Fig. 3 and Fig. S5. [file 40035_2025_519_MOESM3_ESM.pdf]
